# Gene Flow and Hybridization Potential Between GM/NGT Crops and Conventional Varieties or Wild Relatives: A Scoping Literature Review with Emphasis on Oilseed Rape (*Brassica napus* L.) and Potato (*Solanum tuberosum* L.)

**DOI:** 10.3390/biotech15020030

**Published:** 2026-04-08

**Authors:** Lelde Grantina-Ievina, Nils Rostoks

**Affiliations:** 1 The Faculty of Medicine and Life Sciences, University of Latvia, LV-1004 Riga, Latvia; nils.rostoks@lu.lv; 2 Institute of Food Safety, Animal Health and Environment “BIOR”, LV-1076 Riga, Latvia

**Keywords:** environmental risk assessment, genetically modified plants, hybridization, new genomic techniques, oilseed rape, potato, transgene flow

## Abstract

Genetically modified (GM) plants have been commercially grown for 30 years, and their acceptance depends on a thorough risk assessment. Environmental Risk Assessment (ERA) evaluates potential impacts of releasing GM plants into the environment, whether through cultivation or import for food, feed, and processing. A key component is assessing potential gene flow to crop wild relatives or non-GM crops. For gene flow to significantly affect the environment, transferred genes must provide a selective advantage. Since most GM plants are engineered for herbicide tolerance, insect resistance, or stacked traits, evaluating such advantages is relatively straightforward. New genomic techniques (NGTs) can generate plants with a wider range of traits, including tolerance to biotic and abiotic stress. Although still considered GM in the EU, their genomic changes can complicate detection, identification, and ERA, especially when such traits may offer advantages under stress conditions. This scoping review focuses on gene flow in two crops: oilseed rape (canola) (*Brassica napus* L.) and potato (*Solanum tuberosum* L.). In canola, transgene movement can increase weediness, fitness, herbicide resistance, or genetic diversity in feral or related populations. Gene flow in potato is less studied, with concerns centered on contamination risks in the Andean diversity center. Limited data exist for NGT plants, though many are expected to resemble conventionally bred varieties, suggesting comparable environmental impacts.

## 1. Introduction

### 1.1. Introduction to Risk Assessment of GM Plants and Various Plant Modification Techniques

Genetically modified (GM) plants have been grown worldwide for 30 years, with the recent estimate of over 218 million hectares across 31 countries in 2024 [1]. Risk assessment (RA) of GM plants is based on the internationally agreed guidelines, e.g., Codex Alimentarius [2], the Organisation for Economic Co-operation and Development (OECD) “Recombinant DNA Safety Considerations” (Blue Book) [3], and the Cartagena Protocol on Biosafety to the Convention on Biological Diversity [4], while detailed data requirements are laid out in the European Food Safety Authority (EFSA) guidance documents and the European Commission (EC) Implementing Regulation (EU) No. 503/2013 (reviewed in Molitorisová et al. (2024) [5]. RA consists of several steps, including molecular characterization, comparative analysis, food and feed safety assessment, and environmental risk assessment (ERA) and monitoring planning [6,7]. ERA includes assessment of potential unintended effects on plant fitness, potential for gene transfer either from GM plants to environmental microorganisms or to other plants, and interactions with target and non-target organisms or abiotic environment and biochemical cycles. Thus, potential hybridization of GM crop plants with wild species and/or conventional crops is part of the environmental biosafety risk assessment.

The same challenge can be attributed to crops developed by New Genomic Techniques (NGTs). New breeding technologies include such techniques as reverse breeding, cisgenesis, intragenesis, and genome editing [8]. Broothaerts et al. (2021) provide a comprehensive review of NGTs and their implications for various fields, including agriculture, food security, and biotechnology [9]. The review covers several NGTs such as Zinc Finger Nucleases (ZFN-1, ZFN-2, ZFN-3), Transcription Activator-Like Effector Nucleases (TALENs), Meganucleases, clustered regularly interspaced short palindromic repeats (CRISPR) and CRISPR-associated (Cas) systems, and Oligonucleotide-Directed Mutagenesis (ODM). These techniques are used for precise genome editing, enabling targeted gene deletion, replacement, and modification across various species, including plants, animals, and humans. NGTs like CRISPR/Cas are noted for their high efficiency, accuracy, and versatility in genome editing, making them revolutionary tools in molecular biology. Despite their success, NGTs face challenges such as off-target effects, construction problems, and the need for efficient delivery systems for gene editing components. In agriculture, NGTs are used to develop crop varieties with superior qualities and resilience to environmental stresses, which is crucial for addressing food security and climate change challenges [9]. The regulatory status of NGT plants and their products remains a subject of intense discussion around the world, with the options ranging from “allowed for any use” to “not allowed”, while a number of countries, including EU countries, are discussing new legislation for specific types of NGT plants [10].

Cisgenesis and intragenesis are emerging as alternatives to traditional transgenesis in GM plants, and these techniques have been recently risk-assessed [11]. Cisgenesis involves the transfer of genes from sexually compatible species, making it similar to traditional breeding. The environmental risks associated with cisgenic plants are generally considered to be similar to those of conventionally bred plants. The potential for unintended changes, though reduced in comparison to transgenesis, means that each case must be assessed individually. Intragenesis involves the combination of genes and regulatory sequences from the same species or closely related species. As for cisgenesis, intragenesis can result in unintended effects due to random gene insertion. These effects can include disruptions in the host genome and interactions between the inserted gene and the host’s existing genes [11]. The EC has proposed a new regulation that differentiates between two categories of NGT plants: Category 1 (NGT1) and Category 2 (NGT2). NGT1 plants, after a verification procedure, would receive market approval without risk assessment, monitoring, and labeling provisions, while NGT2 plants would require an adapted risk assessment [12,13]. On the one hand, special attention is drawn to complex NGT plant applications, with systemic metabolic changes and plant genotypes that carry multiple mutations within a single generation [14] or that could lead to potentially altered fitness as a cause of new environmental risks [15]. On the other hand, the EC proposal has met criticism as being too conservative, not taking into account the extent of natural variation in plant genomes, ploidy, and genome size [16].

The EFSA guidelines on the environmental risk assessment of GM plants highlight gene flow as a key consideration for persistence and invasiveness, emphasizing that it may either increase the weediness or invasiveness of hybrids or, alternatively, reduce the fitness of wild relatives through effects such as outbreeding depression. The guidance stresses evaluating sexual compatibility, the likelihood and pathways of gene flow (via pollen, seeds, or propagules), and potential consequences such as altered weed dynamics, resistance development, or unintended exposure of non-target organisms. Crucially, gene flow and dispersal do not constitute hazards in themselves. Instead, risk assessment—encompassing both hazard characterization and exposure frequency—and subsequent monitoring must focus on detecting unforeseen ecological consequences associated with GM plant cultivation [6].

### 1.2. Evolution of the Knowledge About the Gene Flow from GM Plants and Hybridization Potential

Gene flow from crops to wild relatives is widespread; spontaneous hybridization across numerous crop–weed complexes enables crop alleles to persist in natural populations and can drive ecological challenges such as the evolution of more aggressive weeds [16]. A well-known example is “weed beet”: Parker and Bartsch (1996) documented hybrids between sea beet (*Beta vulgaris* ssp. *maritima*) and cultivated sugar beet (*Beta vulgaris* ssp. *vulgaris* L.) that formed persistent weed beet populations and significantly reduced European sugar yields [17]. Gene flow has also been observed between GM crops and their non-GM counterparts, including oilseed rape (*Brassica napus* L.), cotton (*Gossypium hirsutum* L.), maize (*Zea mays* L.), soybean (*Glycine max* (L.) Merr.), rice (*Oryza sativa* L.), wheat (*Triticum aestivum* L.), and others [18,19]. For GM oilseed rape, hybridization with wild relatives such as *Brassica campestris* and wild radish (*Raphanus sativus* L.) occurs at low but measurable rates under field conditions and is influenced by distance and pollen dispersal dynamics [20]. Because pollen is the primary vector, high intraspecific gene flow, outcrossing biology, and insufficient containment measures have facilitated extensive transgene escape into both cultivated non-GM and wild relatives [19].

The possibility of gene flow was anticipated even before GM crops were commercialized. Early assessments indicated that hybridization potential varies widely: crops such as sugar beet, carrot (*Daucus carota* subsp. *sativus*), and ryegrass (*Lolium perenne* L. and *L. multiflorum*) have a high likelihood of gene flow due to overlapping ranges and reproductive compatibility, whereas potato (*Solanum tuberosum* L.), maize, tomato (*Solanum lycopersicum* L.), and wheat exhibit minimal hybridization potential outside their centers of origin or diversity [21,22]. For GM potatoes in particular, the risk is considered low because they propagate asexually, and early field studies suggested that an isolation distance of 20 m was adequate during evaluations [20].

Vertical gene flow—transfer of genetic material from parent plants to their progeny via clonal or sexual reproduction—is a natural process, and in genetically engineered (GE) crops (GM and NGT), its success depends on sexual compatibility, pollination conditions, and reproductive isolation mechanisms, all of which may be influenced by genetic engineering [23]. In the 1990s and early 2000s, gene transfer from GM plants to wild relatives was predicted and, in some cases, empirically demonstrated [24]. Contamination through pollen and seed dispersal was identified as the major pathway, prompting recommendations for isolation distances and seed certification protocols to support the coexistence of GM and non-GM crops [25]. Hybridization-mediated transgene escape into wild populations was expected to increase weediness, facilitate habitat invasion, or harm native species interacting with hybrid offspring, thereby posing ecological risks [26].

Many crops—including oilseed rape, barley (*Hordeum vulgare* L.), wheat, beans (*Phaseolus vulgaris* L.), and sugar beet—are capable of hybridizing with their wild relatives [27]. Controlled studies show that wheat, for instance, can hybridize with species such as *Aegilops biuncialis* Vis. at rates ranging from 8.5% to 75%, depending on environmental conditions and cultivar choice [28]. Cross-hybridization requires overlapping flowering periods, with the likelihood increasing as the duration of overlap expands. In the 1990s, however, it was noted that phenological data for wild species were often insufficient: flowering times were known at the country level, but information on habitat-specific variation, genetic diversity, and quantitative parameters was lacking [29].

The subsequent steps in transgene introgression into wild gene pools depend on the formation of hybrid seed and the survival and fertility of hybrid plants. Hybrid infertility can block further transmission of the transgene, while fertile interspecific hybrids grown alongside wild parents typically show low and variable spontaneous outcrossing frequencies depending on the wild species involved [29]. Crop-to-wild gene transfer has been examined in scenarios where interspecific hybridization and even chromosome segment exchange via homoeologous recombination may occur, with studies assessing mechanisms, likelihood, and ecological consequences such as the emergence of invasive weeds or effects on biodiversity [30]. 

A number of factors can determine the spread of a transgene:

(1) Type of transgene: pest or disease resistance, herbicide tolerance, environmental stress (cold, drought, salt) tolerance [31].
(2) Location on the chromosome, which can cause previously unpredictable changes in gene regulation and linkage heritability.
(3) New adaptive traits that the hybrid might possess: resistance to herbicides could confer an advantage in areas where herbicides are used, but not in areas where they are not. Traits related to resistance to diseases and pests could be particularly useful for hybrids of wild plants. Traits such as male sterility and apogamy could have an even more significant impact on seed viability and evolutionary processes. Other important characteristics could be seasonal adaptation, like perennial to annual and daylength response [29,31].
(4) Characteristics of the habitat in which introgression occurs: agricultural land compared to wild habitats.
(5) Genetic structure and size of the recipient wild population and sexual compatibility with it [29,31].
(6) Weediness or invasiveness potential, so-called Baker’s list: “vegetative propagation, self-compatibility, seed dormancy, propagules that are adapted to long-distance dispersal and easily distributed, ability to produce many propagules, ability to germinate in a wide range of conditions, early flowering, strong competitive ability, rapid growth, plasticity of growth, adaptation to disturbed habitats” [31].

Additional factors affecting the establishment of crop–wild hybrids in agricultural or natural habitats were compiled by Dale (1994) (Table 1) [32], who highlighted the importance of biotic pressures such as pests, diseases, and herbivores, as well as a hybrid’s capacity for vegetative and sexual reproduction. Later field experiments in mixed stands of wild plants and first-generation hybrids of *B. napus* and *Brassica rapa* L. confirmed that herbivore presence (pest pressure) can enhance seed production and increase the frequency of *Bacillus thuringiensis* (Bt) plants [33].

For transgene dispersal to occur, three prerequisites must be met: the presence of volunteer plants and/or wild or weedy relatives in or near the cultivated field, pollen dispersal, and seed dispersal. During the establishment phase, these processes may generate escapees, feral populations, or crop–wild hybrids. Subsequent backcrossing, selfing, or sib-mating can lead to introgression and stabilization of hybrid lineages, which may become polyploid or homoploid. The long-term maintenance of transgenes in natural populations depends on the fitness benefits they confer and the genetic costs associated with them [34]. Transgene escape may increase the fitness and competitiveness of wild plants if the trait provides a selective advantage, though effects differ among taxa, environments, and transgenes; some alleles may enhance invasiveness, while others diffuse neutrally without major ecological consequences [35]. The persistence of GM plants as volunteers is therefore a key element in evaluating their invasiveness and weed potential [36]. Successful transgene dispersal further requires successive backcrossing or selfing, recombination between donor and recipient genomes, and introgression of the transgene into the recipient population [37]. The importance of considering recombination rates has been highlighted when assessing the risk of transgene escape. Crops with lower recombination rates, such as sunflower, are less likely to experience transgene escape, while crops with higher recombination rates, such as maize (hotspot), are more susceptible [38].

Early models estimating the probability of gene introgression from GM crops, such as *Brassica* and *Gossypium*, incorporated parameters including adult survivorship, fecundity of wild plants and escapees, seed escape rate, hybridization frequency, polyploid fecundity, hybrid fertility, pollen escape rate, and spontaneous chromosome doubling [39]. A deterministic model for pollen-mediated gene flow in oilseed rape identified target-crop fertility and GM field size as the dominant factors affecting gene flow, showing that isolation distance can reduce gene movement in self-fertile crops but is ineffective for 80% male-sterile non-GM oilseed rape, while large-scale insect transport has minimal influence under conditions of low pollen competition [40].

Conversely, several traits can reduce survival and hinder the establishment of GM escapees, including lack of seed dormancy, reduced pod shattering, uniform maturation, and high seed oil content, which can increase susceptibility to soil pathogens and seed predators [41].

From the late 1990s until around 2010, a large number of field trials were conducted to determine the extent to which gene flow from transgenic plants to conventional varieties was possible [42]. That review examined gene flow from GM plants by summarizing research at that time on mechanisms such as pollen dispersal, seed spread, hybridization, and the persistence of volunteer populations. It also discussed methods to measure and prevent gene flow, emphasizing that while transgenic traits may confer selective advantages, gene flow between crops and wild relatives has long occurred and must be considered in evaluating potential impacts [42].

Scientists in Switzerland have developed the concept of bridge species. Bridge species are wild plant species that facilitate gene flow between crops and their wild relatives or between different wild species. Examples mentioned in the study include species within the *Poaceae* family, such as *Festuca*, *Lolium*, and *Poa*, which are biologically predisposed for hybridization and gene exchange [43].

Hybrids of GM plants and non-GM plants may have increased fitness characteristics. Fitness is a term used in evolutionary biology and refers to an organism’s ability to pass its alleles on to subsequent generations, with survival, growth, and reproductive success used as proxies for quantification purposes [44].

It has been concluded that gene flow from transgenic crops could affect not only the genetic diversity of wild relatives but crop landraces as well, especially in the centers of crop domestication, e.g., Mexico or China [45,46]. A 10% reduction in the abundance or diversity of native wild plant species, compared with relevant comparators, has been proposed as a meaningful effect size for monitoring within a 1 km radius of the cultivation of plants with new traits [47].

In a metapopulation model covering the whole metapopulation of the wild relative, it was calculated that even if the introgression and selection were low, the probability that a transgene will go to fixation in the metapopulation could be high. The species used in the model were annual, diploid, and self-compatible; had no seedbank; and were mating at random [48].

Several molecular strategies for gene containment in transgenic crops were known already in the early 2000s: maternal inheritance, male sterility, seed sterility, cleistogamy, apomixis, genome incompatibility, temporal and tissue-specific control, and transgenic mitigation (TM). Temporal and tissue-specific controls involve using chemically inducible promoters to either activate a gene only when its product is needed or excise the gene before flowering to prevent gene flow. These methods aim to restrict the expression or presence of transgenes to specific developmental stages or tissues, though challenges like incomplete chemical penetration and residual transgene presence remain. TM is a strategy to reduce the fitness of weeds that acquire beneficial traits from GM crops, ensuring that such traits are neutral or advantageous for crops but harmful to weeds. This approach uses tightly linked, non-segregating genes to modify traits such as seed dormancy, shattering, or growth, thereby disadvantaging weeds without compromising crop viability [49].

Possible pre- and post-hybridization transgene management strategies are summarized in Table 2 according to Kwit et al., 2011 [50], although the authors themselves mention several cases in this article where the specific methods of limiting gene spread were not effective. Later developed technologies included female sterility, GeneSafe technologies, parthenocarpy technology, chloroplast transformation, and Gene Deletor technology [51]. It has been demonstrated that hybridization, especially between distantly related species, can activate transposable elements in plant genomes, leading to genomic alterations such as insertions, deletions, and rearrangements. This activation is often triggered by genomic stress during hybridization, contributing to further genetic variation and changes in plant traits [52].

In a review published in 2014, a total of 1783 scientific records on GE crop safety were collected, of which 15% (268) were related to gene flow [53].

Potential vertical gene flow has been studied and modeled in GM plants, which traditionally carry only a few transgenes for specific traits. Newer GM varieties, such as LBFLFK canola with an altered fatty acid profile and eleven newly expressed proteins (NEPs), present additional challenges for environmental risk assessment due to the number of transgenes and possible effects on plant fitness [54]. For NGTs, concerns have been raised that widespread use of CRISPR-edited crops could reduce genetic diversity, promote monocultures, and limit future agricultural adaptability, potentially causing ecological disruptions [55]. These issues apply to all breeding methods, as any approach favoring a few high-performing varieties may replace a broader range of traditional crops. Additionally, multiple genomic changes in NGT plants and resulting novel traits complicate their environmental risk assessment.

This study presents a comprehensive literature review focused on the potential gene flow and hybridization of GM plants and NGT plants with conventional varieties and wild species, particularly highlighting oilseed rape and potato, and is limited to the vertical gene flow. This review aims to assess environmental risks associated with the release of GM crops into the environment and their interactions with non-GM crops and wild relatives. This study also addresses the lack of information on the challenges for the ERA of NGT plants, especially regarding potential gene flow.

## 2. Materials and Methods

### Methodology for the Preliminary Literature Search (Scoping)

The review question Q1 for the preliminary search was as follows: Can genetically modified and NGT plants hybridize with wild plants and conventional varieties? The task was to search for documented cases of genetically modified and NGT plants hybridization with wild plants and/or conventional varieties.

Key elements and synonyms:

- Genetically modified plants, transgenic plants, genetically edited plants, genome-edited plants, CRISPR;
- Hybridization = sexual crossing with viable offspring, outcrossing;
- Wild plants, related species;
- Conventional varieties.

Study designs of interest:

- Experimental studies (greenhouse, mesocosms, in-field);
- Spontaneous hybridization found in nature;
- Previous reviews.

Since publications about potatoes were not discovered using general search string no. 1 (Supplementary Material Tables S1 and S2), the specific review question Q2 for potatoes was defined: Can genetically modified potato hybridize with wild plants and conventional varieties? The task was to search for documented cases of genetically modified potato hybridization with wild plants and/or conventional varieties.

Key elements and synonyms for potatoes:

- Genetically modified potato, transgenic potato, genome-edited potato;
- Hybridization = sexual crossing with viable offspring, outcrossing;
- Wild potato, related species;
- Conventional varieties.

The study designs of interest were the same as those outlined above. The search results are given in Supplementary Material Table S3.

Next, more elaborate search string variants for hybridization in general and potatoes in particular were used in June 2025 (Supplementary Material Table S4). The specific review question Q3 for canola was defined: Can genetically modified or NGT oilseed rape *B. napus* hybridize with wild plants and conventional varieties or establish as volunteer populations? Search strings for NGTs were taken from Eckerstorfer et al. (2019) [56] (Supplementary Material Table S5).

Searches in PubMed (National Institutes of Health (NIH), Bethesda, MD, USA, https://pubmed.ncbi.nlm.nih.gov/, accessed on 9 December 2025) and Europe PMC (European Molecular Biology Laboratory, Cambridge, UK, https://europepmc.org/, accessed on 9 December 2025) using the controlled vocabulary thesaurus of Medical Subject Headings (MeSH, National Institutes of Health (NIH), Bethesda, MD, USA, https://www.ncbi.nlm.nih.gov/mesh/, accessed on 9 December 2025) were used as well (Supplementary Material Table S6).

Additionally, the search term “NGT hybridization risks with other plants” was used in web-based search engine Google Scholar (Google, Mountain View, CA, USA, https://scholar.google.com/, accessed on 9 December 2025). This search returned 775 entries; 50 were evaluated, and 18 were found relevant (36%).

The total number of all search strings (Supplementary Materials Tables S1–S5, results of MeSH in PubMed and an additional search in Google Scholar) resulted in 308,702 literature sources. Due to resource limitations, not all results of the literature search threads were reviewed. In total, 3402 literature sources were evaluated by reviewing the title and abstract. For search results that were above 100, we reviewed 100 items and extrapolated this to the remaining number. This approach was taken from the EFSA guidelines on scoping reviews [57]. If the first 100 were viewed in chronological order, then there was a lot of irrelevant background. If the search tool sorted the sources by relevance, then, of course, there was a better match among the first 100, but this led to an overestimation of the overall matches. In total, the full texts of 724 publications were evaluated. Most of the articles were found in the Europe PMC (228), followed by Web of Science platform Clarivate (Clarivate, London, UK, https://clarivate.com, accessed on 9 December 2025) (149), Google Scholar (146), Scopus database by Elsevier (Elsevier, Amsterdam, The Netherlands, www.elsevier.com/solutions/scopus, accessed on 9 December 2025) (123), and PubMed (103). A total of 537 of these entries were rated as relevant, i.e., the information was relevant to the search query and the keywords could be found in the article. Some (237) were included in the review (PRISMA flow diagram for the searches of databases is included in Supplementary Material Figure S1) [58]. Generative artificial intelligence (GenAI) of Scopus AI embedded in Scopus (Elsevier, Amsterdam, The Netherlands) and Acrobat AI Assistant (Adobe, San Jose, CA, USA) was used to summarize the results of 88 publications. The AI tools were tasked with searching for specific information in a particular publication. The results were manually reviewed by experts. In addition, expert knowledge was used to add some relevant publications.

## 3. Results

### 3.1. Analyses of the Preliminary Literature Search (Scoping)

The results of preliminary searches in several databases by combining variations of the key elements of the review question Q1 with AND are given in Supplementary Material Table S1 (searched in September 2024). Good compliance with the research question was shown for search string no. 1 in the Web of Science database: out of 177 literature items, 129 were identified as appropriate for the research question through expert evaluation (72.88%). The most frequently described hybridization cases by plant species, family, etc., in the literature according to search term no. 1 “Genetically modified plants AND hybrid AND wild plants” in the Web of Science database (n = 126) are given in Supplementary Material Table S2. Publications about potatoes were not discovered using general search string no. 1.

The results of preliminary searches in several databases by combining variations of the key elements of the review question Q2 about potatoes with AND are given in Supplementary Material Table S3 (searched in June 2025).

Next, more elaborate search string variants for hybridization in general and for potatoes and canola in particular were searched in June 2025, and the results are given in Supplementary Material Table S4.

The results of the search strings for NGTs from Eckerstorfer et al. (2019) [56] are given in Supplementary Material Table S5.

The key outcomes of all the search strings are summarized in Table 3.

### 3.2. Analyses of Search Results on Brassicaceae Species

#### 3.2.1. The Role of Soil Seed Bank

In the case of *Brassica* cultivated crops, their origin is quite complicated, with several cultivated species being allopolyploid hybrids:

(1) Field mustard *B. rapa*—AA (2n = 20);
(2) Black mustard *Brassica nigra* (L.) W.D.J.Koch—BB (2n = 16);
(3) Broccoli *Brassica oleracea* L.—CC (2n = 18);
(4) Chinese mustard *Brassica juncea* (L.) Czern.—AABB (2n = 36);
(5) Ethiopian mustard *Brassica carinata* A. Braun—BBCC (2n = 34);
(6) Canola *B. napus*—AACC (2n = 38) [59].

Oilseed rape is self-compatible, with outcrossing rates ranging from 5% to 55%, and its seeds can persist in the soil seed bank for at least 10 years. The soil seed bank refers to the reservoir of viable seeds that accumulate and persist in soil, sometimes for many years, and can later germinate to produce plants. In agriculture, this includes seeds from crops, volunteers, or wild relatives. For oilseed rape, spilled seeds may enter dormancy and survive for over a decade, leading to volunteer plants in future seasons. This persistence is significant for understanding gene flow and the long-term presence of GM varieties in fields [60,61]. The findings in Denmark indicated that the plants or seeds originated from non-transgenic varieties grown at the site 4–17 years prior, demonstrating prolonged persistence of volunteer populations. Substantial volunteer incidence (6%, 29%, and 32%) was recorded across the three oilseed rape fields examined, and soil core analyses estimated a seed bank density of approximately 50–100 seeds per square meter [62].

Farm-scale GM trials established in France in 1995 were utilized to assess the presence of GM seeds in the harvest of conventional varieties planted 3–8 years later on the same fields. The results indicate that over time, GM seed admixture in the harvest frequently exceeded the European threshold (0.9% for admixtures), occurring in 6 of 18 instances, with one case reaching a level as high as 18% [63].

There is more information in the literature that field trials of GM plants can lead to the formation of the soil seed bank and the periodic appearance of GM plants in the environment. From 1996 to 2001, field trials were conducted in the Saxony-Anhalt region of Germany with various GM oilseed rape types, including winter oilseed rape varieties. Then, 15 years after the end of the field trials, monitoring was carried out at the field trial site, and DNA analyses were performed on the various oilseed rape samples found. The presence of GM oilseed rape was detected in individual cases, which confirmed the ability of oilseed rape seeds to persist in the soil for a long period of time, but the formation and spread of stable populations was not observed. There was no spatial dispersal or stable establishment of GM oilseed rape populations in the environment surrounding the former field trial sites over the 15-year monitoring period. The findings indicate that GM oilseed rape did not become invasive or form stable populations outside cultivated and ruderal habitats. This conclusion aligns with other observations that feral oilseed rape populations, including GM varieties, are typically associated with repeated cultivation or spillage during transport rather than long-term environmental establishment [64]. During the same time period, USA scientists developed criteria for field testing of plants with some particular traits. The main criteria for field trials with GM canola included ensuring confinement to prevent gene flow, such as using isolation distances, monitoring for volunteers, and employing seed-specific promoters to focus risk evaluation on seed traits. Additionally, regulatory standards required stable gene integration, known gene function, and no production of toxic substances or infectious entities [65]. In a study in the United Kingdom, transgenic lines of oilseed rape (*B. napus*) showed lower seed survival compared to non-transgenic lines, with survival rates of 0.3% for kanamycin-tolerant seeds and 0.25% for seeds tolerant to both kanamycin and glufosinate after the first year. The study found no significant difference in survival between the two transgenic lines, suggesting that the additional genetic traits did not increase persistence [66].

#### 3.2.2. The Evidence from Natural Conditions and Spontaneous Outcrossing

In the early 2000s, some scientists reasoned that gene flow between crops and their wild relatives is common and inevitable when grown within the crop’s pollen dispersal range, as hybridization occurs naturally between compatible plants. Canola was considered as one of the out-crossing species, along with corn and sunflower, where gene flow to non-transgenic crops of the same cultivated species is a real concern. Scientists noted that gene flow from crops like canola to non-transgenic crops or wild relatives is possible, especially due to their outcrossing nature. This raised issues such as the potential for “genetic contamination” of organic crops and the risk of patent violations if transgenes are found in neighboring fields. They highlighted that regulatory frameworks often restrict the planting of transgenic crops in areas where wild relatives are endemic to limit the risk of genetic contamination and the creation of “superweeds” with enhanced survival traits. Additionally, they emphasized the importance of monitoring and containment strategies, such as border rows, trap crops, and fallow zones, to mitigate the ecological risks associated with gene flow [24].

Herbicide-tolerant volunteers and weeds have been ranked as the highest risk factors for ecological hazards [67]. Wild Brassicaceae species have self-incompatibility systems [68] that allow the transgene to spread rapidly within a population. Gene flow and gene drift must also be considered. Spread to subsequent populations depends on the distance between them, plant density, pollination type, presence of pollinators, population size, and genotypic composition [29]. In classical oilseed rape breeding, it has been observed that if there is a distance of 100 m between two oilseed rape varieties, then this ensures 99.9% purity of the variety. Oilseed rape and related weeds produce many times more pollen than ovules, so there is a lot of competition for pollen on the stigma. It should be remembered that there are two different processes: pollen dispersal and actual pollination [69].

A study in China assessed the risk of gene flow from canola (*B. napus*) to its sexually compatible wild relatives, primarily *B. juncea* and *B. rapa*, by integrating GIS-based cultivation data with MaxEnt species distribution modeling. It found that gene flow risk exists across much of the country—especially in regions with high wild *Brassica* richness—and is of particular concern because China is considered the center of origin for *B. juncea* and *B. rapa*, meaning that introgression could negatively affect the conservation and genetic integrity of local biodiversity, prompting recommendations for targeted monitoring and regional management [70].

In one of the review articles, the authors explained that gene flow from GM *B. napus* (canola) to non-GM or related weedy species primarily occurs through pollen and seed dispersal, with the highest hybridization rates observed within the first 10 m of the recipient field. Empirical investigations indicated that the average gene flow rate (1.78%) was greatest in immediate proximity to the pollen source and declined to approximately 0.05% within the first 10 m. In experiments employing a discontinuous spatial design, gene flow exhibited a gradual attenuation with increasing distance. It remained relatively stable adjacent to the source, with a mean value of 0.94%, and persisted at an approximately constant level of 0.1% up to 100 m. They suggested strategies to mitigate gene flow, such as establishing buffer zones, using cleistogamous varieties, and selecting appropriate recipient plants or herbicides [71].

In some countries, it has been evaluated which wild *Brassicaceae* species could potentially hybridize with transgenic *B. napus*. For example, in Flanders, Belgium, the highest introgressive hybridization propensity was estimated for *B. rapa*, *Hirschfeldia incana* (L.) Lagr.-Foss., *Raphanus raphanistrum* L., *B. juncea*, *Diplotaxis tenuifolia* (L.) DC., and *Sinapis arvensis* L. [72]. In Canada, some field experiments were carried out showing that hybridization between transgenic *B. napus* and its wild relative *B. rapa* occurred at notable frequencies in two field experiments (approximately 7%) and commercial fields (up to 13.6%), with hybrids showing reduced pollen viability and a triploid genome. In contrast, hybridization with other wild relatives like *R. raphanistrum*, *S. arvensis*, and *Erucastrum gallicum* (Willd.) O.E.Schulz was extremely rare or undetectable, indicating a very low probability of gene flow to these species [73]. In Canada, it has been observed that weedy *B. napus* and *B. rapa* exhibit herbicide resistance, with *B. napus* having transgenic resistance to glyphosate and glufosinate and mutagenesis-derived resistance to imidazolinones. Herbicide resistance in *B. rapa* was maternally inherited, and transgene escape from *B. napus* to *B. rapa* has been documented, leading to hybrid populations with stacked resistance traits [74].

The summarized information about reported cross-pollination frequencies at specific distances and maximum distances where gene flow was detected is given in Table 4. Most realized cross-pollination in oilseed rape occurs within tens of meters; frequencies > 1% are generally within ~10–30 m. Rare events can occur at much longer distances [75] (Liu et al., 2013). Fully fertile *B. napus* seed crops with ~100 m separation typically maintain cross-pollination below ~0.5% for most conditions; 200–400 m can push frequencies toward ≤0.1% in small plot standards. Varieties containing male sterile components or varietal associations may require larger separations than fully fertile varieties [22] (Eastham/ESF review 2002). Species pairs differ: *B. napus* ↔ *B. rapa* hybridization is the most frequently observed interspecific route; *B. juncea* and *B. carinata* show much lower rates that nonetheless extend to 150–400 m in production mosaics [76] (Lal 2020). Interspecific hybrids with wild radish occur at very low frequencies, with the highest rates at field borders [20] (Messeguer 2003).

#### 3.2.3. The Formation of Hybrid Seeds and the Survival of Hybrids

As already mentioned, an important step in the introgression of transgenes into the gene pool of wild species is the formation of hybrid seeds and the survival of hybrids. In an experiment conducted in the 1990s, when crossing field mustard (*B. rapa*) with GM oilseed rape (*B. napus*) T151 and T152, which were resistant to glufosinate herbicides, only 98 hybrid seeds were obtained from 2000 pollinated flowers, which the authors considered to be a relatively high number, about one hybrid seed for every 25 pollinated flowers. Plants obtained from these seeds were 97% resistant to the respective herbicide, but only two of these progenies (approx. 2%) were able to produce seeds that were capable of self-pollination; however, they could be successfully pollinated by both field mustard and oilseed rape [81]. In other studies, the seed production of F1 hybrids between *B. rapa* and *B. napus* has been recorded as high [82,83,84,85]. On average, F2 generation and backcrosses show reduced fitness in comparison to their parents, but some individuals were as fit as the parents [83,86]. In a study of four generations of intergeneric hybrids between glufosinate-tolerant *B. napus* and such weedy species as hoary mustard (*H. incana*), wild mustard (*S. arvensis*), and wild radish (*R. raphanistrum*), it was shown that the transgene transmission was reduced but the fertility was increased [87]. Similar results were obtained in a study with transgenic oilseed rape and *B. rapa* (collected in Denmark) in BC3 plants [88]. In another study with *H. incana,* it was observed that there is no post-zygotic barrier to the development of hybrid embryos [89].

However, hybridization under natural conditions in the United Kingdom has been recorded: one hybrid between non-GM *B. napus* and *B. rapa* among 505 plants screened in the *B. rapa* populations and zero hybrids between non-GM *B. napus* and *B. oleracea* among nine newly established plants screened in the *B. oleracea* population for hybrid status [90,91]. Nevertheless, the authors developed the satellite image-processing method for countrywide remote sensing of oilseed rape fields and recipient riverside *B. rapa* populations [92]. In field experiments carried out in the USA, Bt-transgenic oilseed rape varieties were planted together with *B. rapa* as a female parent at a ratio of 1200:1. The detected hybridization frequency ranged from 16.9% to 0.7%, and the resultant hybrids synthesized the Bt Cry1Ac protein at similar levels as the transgenic parents. Transgenic *B. rapa*-like plants could be obtained in three further generations with backcrossing [93]. A further study demonstrated that transgene flow from transgenic oilseed rape (*B. napus*) to its wild relative *B. rapa* occurred under various field conditions, with hybridization frequencies ranging from approximately 2% to 37.2%, depending on the crop-to-wild relative ratios and proximity within the field. No hybridization was detected between transgenic oilseed rape and *R. raphanistrum*, and transgene backcrossing frequencies from hybrids to *B. rapa* were low, averaging 0.088% and 0.060% across two experimental locations [94].

Still, the knowledge of the occurrence of hybridization between GM oilseed crops and wild related plants and hybrid establishment in nature 20 years ago was evaluated as insufficient [95]. Although the gene flow and hybridization for plants, including hybridization between crops and their wild relatives, were already well documented at that time [96]. In his review [96], N.C. Ellstrand formulated six conclusions about the gene flow:

(1) it is not unusual for crops to mate with their wild relatives;
(2) gene flow, in itself, does not necessarily create problems;
(3) natural hybridization occasionally results in problems in terms of increased weediness or invasiveness;
(4) natural hybridization occasionally results in negative impacts in terms of increased extinction risk to wild relatives;
(5) gene flow varies tremendously, both between species and within species;
(6) typically, intraspecific gene flow occurs at surprisingly high rates and over surprisingly high distances. Multiple herbicide-resistant *B. napus* plants were detected in Alberta (Canada) more than 550 m away from the pollen source 1.5 years after seeding [96].

In a field study in New Zealand using hand pollination, the hybridization rate between fully fertile non-transgenic herbicide-resistant *B. napus* and wild turnip (*B. rapa* var. *oleifera*) was 41% of all pollinated stigma. Almost 100% of progenies were herbicide-resistant [97].

In one study by Canadian and USA scientists, Bt-transgenic *B. napus* and *B. rapa* hybrids were grown in hydroponic, glasshouse, and field experiments, and the investigations resulted in hybrid populations being less productive and competitive [98].

Spontaneous hybridization between transgenic male-sterile oilseed rape (*B. napus*) and wild radish (*R. raphanistrum*) as well as cultivated radish (*Raphanus sativus*) has been observed. The frequency of hybrids varied significantly among populations with different origins (from various countries), with some showing high hybridization rates [99].

Hybridization between glyphosate-resistant *B. napus* and its weedy relative *B. rapa* was monitored over several years in Canada. The persistence of the herbicide resistance transgene in *B. rapa* populations was confirmed, indicating stable introgression [100]. One study of field trials in Canada found that 52% of locally grown rapeseed lots exhibited resistance to glyphosate and glufosinate above 0.25%, falling short of the 99.75% seed purity standard. The authors ruled out pollen as the source of contamination, since growers maintained recommended field separation distances, and instead suggested that mechanical seed mixing during harvest or contamination from earlier plant generations was likely responsible [101]. Further evidence comes from international studies that have traced GM oilseed rape contamination back to Canadian imports. For instance, research in Switzerland found that GM oilseed rape seeds were present in wheat imports from Canada, concluding that even non-GM crops can carry traces of GM seeds due to contamination [102]. Seed contamination can later lead to hybridization.

In a large-scale study carried out in Germany, it was empirically observed that feral oilseed rape *B. napus* plants were, on average, at least 41% smaller than cultivated oilseed rape after the flowering stage [103]. Another study found that transgene persistence decreased significantly in hybrid populations containing TM constructs. Specifically, the frequency of transgenic individuals in TM hybrid progeny dropped dramatically (e.g., less than 1% in competitive conditions) compared to non-TM hybrids, which showed only a threefold reduction in transgene persistence. This demonstrates that TM strategies, such as incorporating a dwarfing gene, effectively limit transgene persistence in weedy backgrounds [104]. 

Smaller hybrid seed size under field conditions exhibited lower frequency of emergence, reduced seedling survival, later flowering, and smaller plant biomass in comparison to plants developed from larger seeds [105]. While hybrids initially can face reduced fitness and fertility, successive generations may stabilize, allowing transgenes to persist and impact agricultural practices or ecological dynamics [75]. The Bt-transgene may not provide an advantage in mixed stands of backcrossed hybrids, meaning its introgression would not be promoted in the absence of herbivorous insects. However, if there is a relatively large initial population of Bt-transgenic plants, the transgene could persist when target herbivores are present [106]. In a greenhouse experiment, it was observed that F1 hybrids between GM *B. napus* and non-GM *B. rapa* exhibit intermediate traits, such as seed size, stomatal density, and fatty acid composition, with erucic acid levels being between those of the parent species. They flower slightly later than transgenic *B. napus*, show abnormal pollen morphology (~40%), and cannot produce F2 progeny but can generate BC1 progeny through backcrossing with *B. rapa* [107]. 

In a four-year-long field survey in northwest Germany, 78 sites with non-GM feral oilseed rape populations were found and monitored. The seed production rate was quite high, with 30–48% of feral populations producing seeds, and the genetic diversity of the plants in these feral populations was higher than that of cultivated varieties in these regions [108].

One of the first cases in the world where ruderal oilseed rape populations have been observed to cross with GM oilseed rape for a long time, over a 10-year period, was found in Japan. Ruderal oilseed rape plants on the side of the highway were resistant to both glyphosate and glufosinate. Offspring from plants whose seeds were collected on the side of the highway were resistant to these herbicides, and they also contained sequences corresponding to GM oilseed rape with high similarity to known GM oilseed rape sequences: *cp4 epsps* and *bar* [109,110,111]. Furthermore, large plant individuals of feral GM oilseed rape in Japan exhibited perennial growth under natural conditions, which is not observed in domestic *Brassicaceae* species. The authors concluded that this perennial growth increases the likelihood of gene transfer to domestic and wild relatives, contributing to the persistence and spread of GM traits in the environment [112]. 

Another well-documented case was first found in Canada in 2001 in two locations [100,111], where introgression of herbicide tolerance in *B. rapa* was demonstrated and persisted over a six-year period [100,113]. Other cases of GM oilseed rape growing in the environment were documented in the USA, Switzerland, and Argentina between 2008 and 2012 [111]. In a study in the USA, it was found that GM canola populations have extensively escaped cultivation in North Dakota, with 80% of sampled roadside populations expressing transgenes for herbicide resistance. These feral populations were widespread, persistent, capable of hybridizing to produce novel genotypes, and likely established through mechanisms such as seed spill during transport and reseeding from soil seed banks [114]. In Switzerland, GM oilseed rape hybrids with conventional plants were found as well [115]. Insect-resistant oilseed rape *B. napus* containing the *Cry1Ac* gene for insect resistance and *B. rapa* hybrids were obtained in an outdoor mesocosm study in the USA, and, notably, the plants were larger, with more seeds than the weedy parent [116].

In an experiment under field conditions in the USA, it was observed that transgenic genotypes of hybrids between herbicide-resistant *B. napus* and weedy *B. rapa* showed increased plant fitness resulting from glyphosate-drift treatments [117]. In controlled conditions in France, herbicide-resistant BC1 plants (progenies of herbicide-resistant *B. napus* and wild *B. juncea*) showed *B. napus* morphology and had larger flowers and high genetic variability [118].

In the 2010s in Europe, scientists considered concerns about feral genetically modified herbicide-tolerant (GMHT) oilseed rape and its potential to introduce herbicide tolerance traits into agricultural or semi-natural habitats, leading to environmental or economic issues such as increased weed problems or GM admixtures in non-GM crops. However, scientific evidence suggested at that time that feral GMHT oilseed rape is unlikely to become invasive or significantly impact coexistence thresholds, and routine management may not be necessary [119]. 

One of the first countries in Europe to monitor for the presence of wild populations of GM oilseed rape was Switzerland. In this country, cultivation of GM crops has never been authorized, and accidental spillage of GM seeds was considered an undesirable effect of GM plant material import. In 2014, Swiss scientists published two articles on the occurrence of wild oilseed rape along transport routes and at processing sites in the country. The study was conducted in 2011 and 2012. The aim of the study was to carry out risk-based monitoring of GM oilseed rape along railway lines from the Swiss border with Italy and France to oilseed rape processing plants. Two oilseed rape processing plants and the railway sections used to supply them in the south and north of the country, 37 and 15 km long respectively, as well as two transshipment points in the Rhine River port of Basel, were selected for monitoring. Railway crossings and sharp bends were considered as hotspots for the spillage. The glyphosate-resistant GT73 oilseed rape (Roundup Ready, Monsanto) was found in three locations: Lugano station, the Rhine port near Basel, and the border crossing point St. Louis–St. Johann station–tunnel entrance Kannenfeldplatz. It should be noted that railway tracks in Switzerland are regularly treated with glyphosate-containing herbicides. The GM oilseed rape plants found contained the glyphosate oxidoreductase gene *gox* and CP4 *epsps* genetic elements and were resistant to glyphosate. Glufosinate-resistant GM events MS8xRF3, MS8, and RF3 (InVigor, Bayer) were detected for the first time in Europe at five locations in the Rhine port of Basel. They contained the *bar* gene conferring resistance to glufosinate. None of the plants of the potential hybridization partner species collected were GM [115,120].

Hybrids between *B. napus* (AACC) and *B. rapa* (AA) were found in field conditions in several other European countries as well: the UK, the Netherlands (only F1 hybrids), and Denmark (F1 and backcross plants). In the Netherlands, hybrids were found in mixed populations and were more commonly located on the roadsides [121]. Evaluating these data with various model tests, other scientists from the Netherlands suggested that the selection is against unpaired C-chromosomes [122]. This principle was later proven by Chinese scientists, who observed that C-genome-specific markers were lost faster [123]. In a later study, it was found that although most C-genome-specific genomic regions were extensively eliminated, the glyphosate resistance gene from the male parent *B. napus* was successfully introgressed into BC3 progenies, indicating that the gene is located and integrated within the A-chromosome/genome regions of the *B. rapa* plants [124]. 

In a different modeling study, it was found that if the population of wild relative radish (*R. raphanistrum*) is viable and enlarging the scope of transgene dispersal, then the risks for transgene dispersal from *B. napus* within such a population are quite certain in comparison to reducing the population of the wild relative [125]. It was observed earlier that interspecific hybridization depends on the genetic structure of the *R. raphanistrum* population [126].

Ten years ago, oilseed rape was mainly imported into Europe through Dutch and Belgian ports, while Hamburg was a less significant importer. In Germany, river transport and unloading at ports and processing sites posed spillage risks. Plants near the Rhine were surveyed in spring 2014. Wild oilseed rape was widespread; wild crucifers (e.g., *S. arvensis*) were rare. Most oilseed rape plants were observed and sampled along roads and railway tracks. In most sample locations (55%), 6 to 25 individuals were present, but groups of plants up to 50 individuals were also relatively common (21%). Massive oilseed rape populations (up to 500 individuals) were observed in individual ports. During the spring period, 136 samples from 1702 plants were collected at 9 study locations, and only one plant turned out to be transgenic (glyphosate-resistant). During the summer, seeds from 216 plants were collected in 20 sample plots near the GM oilseed rape discovery site in the port of Neuss, obtaining 1918 plants for further analysis, but no transgenic plants were detected. Oilseed rape seeds spilled on roadsides may germinate in the current growing season but may also enter a secondary dormancy period and form a soil seed bank, with possible germination within at least 3 years, but possibly up to 15 years. The fact that only one GM oilseed rape plant was found in the study could be due to the fact that the random sampling method used in the study did not fully represent the total wild oilseed rape population. This type of monitoring should be carried out regularly near potential contamination sites, with detailed studies carried out in places where GM oilseed rape samples are found [127].

In order to understand the potential of hybrid formation between transgenic plants and their wild relatives, it is important to know some typical characteristics of the wild relatives, like production characteristics (pods per plant, seeds per plant, seeds per pod, etc.) [128] and preferences of natural pollinators [129], as well as the maximum distance for wind pollination [130].

Other *Brassicaceae* species studied for hybridization include hybrids between transgenic broccoli *B. oleracea* and non-transgenic broccoli, kale, and cauliflower. In one study, broccoli was transformed to have an isopentenyl transferase *ipt* gene to delay senescence. Pollen viability and pod setting rate were evaluated. In crosses with transgenic broccoli, higher pollen viability was observed, as well as larger numbers of pollen and their grain size, and higher pod setting rates [131].

One of the last documented cases of hybridization between GM canola and wild plants was in Argentina, where imidazolinone-resistant wild turnip *B. rapa* × *B. napus* hybrids and GM glyphosate-resistant *B. rapa* were detected in field conditions [132,133]. In an experiment, scientists found no significant differences in fitness-related traits, such as plant height, seed production, and pollen viability, between herbicide-resistant and susceptible *B. rapa* populations, indicating that the resistance traits do not negatively impact the plants’ reproductive or vegetative performance. This suggests that the glyphosate resistance transgene and imidazolinone-resistant mutation can persist in the environment without selective herbicide pressure, as they do not impose a fitness cost [133].

In a comprehensive review article [134], the authors conclude that interspecific hybridization between transgenic *B. napus* and *B. rapa* can successfully occur in controlled environments, such as greenhouses and experimental fields, producing fertile and viable generations that pass herbicide-resistant transgenes to their offspring. Artificial hand pollination in greenhouse conditions results in a 100% outcrossing rate, while spontaneous hybridization under field conditions has a significantly lower rate due to external factors. The authors highlight the environmental risks of transgene persistence in nature, including gene flow to wild relatives, increased weediness, and reduced fitness in hybridization and introgression. They suggest strategies to mitigate transgene spread, such as greenhouse containment, integration into the C genome, and advanced genome editing technologies. Future research directions include leveraging genome sequencing, phenomics platforms, machine learning, and genome editing to develop improved crop plants with traits that help control transgene spread, such as altered flower or leaf colors for easier identification and elimination of transgenic volunteers or weeds [134].

In a recent study in Canada, scientists analyzed the genetic characterization and distribution of glyphosate-resistant bird rape mustard (*B. rapa*) populations, focusing on the introgression of glyphosate resistance from GT73 canola (*B. napus*). The introgression of the GT73 transgene was detected. The ease of hybridization between *B. napus* and *B. rapa* facilitates the transfer of glyphosate resistance. This gene flow can occur in field borders or areas where the two species co-occur, leading to the spread of resistance in wild populations. Glyphosate-resistant *B. rapa* populations are difficult to control in fields where glyphosate is heavily used, such as in glyphosate-tolerant corn and soybean rotations. This resistance can lead to uncontrolled weed populations, reducing crop yields and increasing management costs. The study also identified glyphosate resistance in *R. raphanistrum*, suggesting that transgenes could escape into other weedy species, further complicating weed control efforts [113].

In a follow-up study in the USA, feral transgenic canola populations with herbicide resistance were found in 2021 to be established outside cultivation fields in an unmanaged environment. Feral canola plants had *CP4 EPSPS* and/or the *PAT* gene [135]. A similar situation was documented in South Korea, where GM canola plants were detected for over 15 years along roadsides, feed factories, and festival areas [136]. The key difference between the two countries is that the USA grows GM canola, while South Korea only imports it.

Recently, the monitoring conducted in Italy focused on assessing the accidental dispersal of GM oilseed rape seeds, specifically in the Campania region. ISPRA and ARPA Campania developed and tested a monitoring protocol that included transect identification, sampling, and DNA analysis. Field surveys were carried out along two routes (Salerno–Benevento and Salerno–Caserta) between 2018 and 2019, targeting hotspots such as the port of Salerno and seed companies. The study identified interfertile species like wild radish (*R. raphanistrum*) near industrial sites, supporting the hypothesis of potential gene flow, although the DNA analysis confirmed the absence of GM material in the samples. The protocol will be applied to strengthen post-market environmental monitoring (PMEM) in Italy, focusing on high-risk areas such as entry points, storage sites, and transportation routes. The findings will support the implementation of national surveillance activities and stricter measures to minimize seed dispersion during transport and handling of GM seeds [137]. 

Documented cases and potential occurrence and persistence of transgenic oilseed rape populations in various ruderal habitats across multiple countries are given in Table 5.

A review study to estimate pollen-mediated gene flow at the landscape scale of oilseed rape and other crops (maize, rice, and wheat) summarized diverse factors that can influence the gene flow process (Table 6).

New canola characteristics could bring some new challenges for environmental risk assessment, including gene flow. For example, there are two seed oil crops, *Camelina sativa* (L.) Crantz (false flax) and *B. juncea*, expressing microalgal genes with altered profiles of polyunsaturated fatty acids. The genes modified for engineering DPA and DHA in *Brassica juncea* included Δ12-desaturase (Δ12-Des), ω3-desaturase (ω3-Des), Δ6-desaturase (Δ6-Des), Δ5-elongase (Δ5-Elo), Δ5-desaturase (Δ5-Des), Δ4-desaturase (Δ4-Des), and Δ6-elongase (Δ6-Elo). These enzymes are part of the aerobic long-chain polyunsaturated fatty acids biosynthesis pathway introduced into the crop [152,153].

#### 3.2.4. Conclusions About Brassicaceae

The previous Section 3.2. delved into the complex origins of cultivated *Brassica* species, highlighting their allopolyploid hybrid nature. It discussed the gene flow and hybridization potential between GM oilseed rape (*B. napus*) and its wild relatives. The findings indicate that hybridization between GM oilseed rape and its wild relatives can occur, with documented cases of gene flow and hybrid establishment in various regions. Key points included:

1. Hybridization Potential: Theoretically, GM oilseed rape can hybridize with wild relatives like *B. rapa*, *H. incana*, *R. raphanistrum*, *B. juncea*, *D. tenuifolia*, and *S. arvensis* [65]. Field experiments in Canada showed notable hybridization frequencies between transgenic *B. napus* and *B. rapa* [73].
2. Persistence and Spread: GM oilseed rape seeds can persist in the soil seed bank for at least 10 years. Monitoring in Germany revealed the presence of GM oilseed rape 15 years after field trials [59]. In Japan, GM oilseed rape with multiple herbicide resistance was found along roadsides [109,110,111,112].
3. Environmental Impact: Hybridization can impact weediness [26,72] and increase fitness in the presence of herbicide applications [73,74,111,112], herbicide resistance [85], and genetic diversity of feral oilseed rape populations [104] or wild relatives [113], all as a result of transgene movement [87]. The persistence of herbicide resistance in wild *B. rapa* populations was confirmed in Canada. In several studies, it was concluded that potential hybridization of transgenic *Brassicaceae* with wild species and/or conventional crops is one of the environmental biosafety risk factors [6,97].
4. Regulatory and Monitoring Efforts: Various countries conducted studies to monitor and assess the spread of GM oilseed rape. For example, Switzerland detected GM oilseed rape along railway lines [102,115,120] and Italy developed a monitoring protocol for accidental dispersal [137].
5. Mitigation Strategies: Strategies to mitigate gene flow include using isolation distances, monitoring for volunteers, and employing seed-specific promoters [65]. Transgenic mitigation (TM) constructs have been shown to reduce transgene persistence in hybrid populations [49,104].

Despite this substantial body of evidence, several important knowledge gaps remain. First, the long-term ecological dynamics of hybrids and introgressed populations remain insufficiently understood, particularly under varying environmental conditions and management regimes. Second, there is limited quantitative information on the fitness and persistence of hybrid populations in the absence of selective pressures, such as herbicide applications. Third, regional differences in gene flow dynamics, especially outside intensively studied areas such as Canada, remain poorly characterized. Finally, while monitoring programs have documented the occurrence of feral populations and transgene escape, harmonized long-term monitoring frameworks and standardized methodologies are still lacking.

Addressing these gaps through coordinated field studies, long-term monitoring, and improved ecological modeling would help refine environmental risk assessments and support evidence-based regulatory decision-making for oilseed rape and related crops.

### 3.3. Analyses on Search Results on Potatoes

#### 3.3.1. Potato Genetics and Breeding History of GM Potatoes

Research on potato genetics and breeding has focused on germplasm enhancement, utilization of wild relatives, and breeding strategies for improved traits. Advances in biotechnology and genomics are reshaping breeding approaches, allowing for more precise selection and gene discovery.

Sexual hybridization in potato is biologically possible. Cultivated potato (*S. tuberosum*) and many wild relatives in the *Solanum* (section *Petota*) group are sexually compatible to varying degrees; interspecific crosses and introgression have been documented and are used in breeding. That underlying biological compatibility means that gene flow can occur where flowering and pollination happen [154].

Most of the cultivated potato varieties are tetraploid (2n = 4x = 48), although in the Andean region of South America, triploid and pentaploid cultivated species have been grown. Common tuber-bearing *Solanum* spp. cultivars have poor flowering and male sterility, leading to no cross-hybridization or unilateral hybridization, respectively [155]. Male sterility in the tetraploid 4x-2x hybrids could be beneficial in reducing outcrossing to sexually compatible wild potato relatives. This could potentially mitigate the risk of transgene escape into wild populations, which is a common environmental concern associated with GM crops [156].

The genetic engineering of potatoes began in the late 1980s and 1990s, with the International Potato Center collaborating with institutions like Cornell University and Wageningen University to explore transgenic approaches for introducing traits not available in existing germplasm. Early efforts focused on inserting target transgenes to accelerate breeding while minimizing undesired genetic variation, alongside risk assessments for environmental and food safety under the Cartagena Protocol on Biosafety. GM potatoes were created and approved for cultivation in the USA and Canada in the 1990s, including virus-resistant varieties. The two most important potato viruses that were addressed with these GM lines were the potato leaf roll virus and the potato virus Y [157].

In a study in the Netherlands in 1997, scientists successfully produced somatic hybrids between *S. tuberosum* and species of the *Solanum nigrum* complex, with the ploidy level and parental genotype significantly influencing somatic combining ability and hybrid viability. Among the 16 fusion combinations tested, only three—*ngr* (+) 1029/31, *ngr* (+) AM10K, and *ame*_2x_ (+) Desirée—yielded a substantial number of vigorous and flowering hybrids suitable for backcross experiments [158].

Cisgenesis enabled the stacking of resistance genes directly from the same species into potatoes without introducing foreign DNA, preserving the plant’s genetic integrity. It offered a faster and more efficient alternative to introgression breeding by avoiding linkage drag and ensuring compatibility with natural breeding processes [159].

Today, transgenic potatoes with enhanced traits such as stress tolerance and pest resistance are approved for cultivation in some countries, though a broader consensus on biosafety and application remains a challenge [160]. Examples include transgenic potatoes expressing genes like *cry1Ab* for insect resistance, OC-IDD86 for nematode resistance, and StnsLTP1 for enhanced tolerance to heat, drought, and salinity [161]. Resistance to late potato blight is a significant target, with some research studies showing potential breakthrough through the use of transgenes or NGTs [162,163,164,165]. Non-lethal markers and marker-free systems for potatoes include the use of co-transformation methods, such as *Agrobacterium*-mediated transformation (AMT) without selectable markers, and site-specific recombination systems like Cre/loxP for marker gene excision. These approaches have successfully produced marker-free transgenic potatoes with traits like oxidative stress tolerance and amylase-free characteristics, demonstrating efficient transformation and regeneration [166]. Researchers successfully developed selectable marker-free transgenic potato plants expressing the Cry3A protein using Agrobacterium-mediated cotransformation and self-crossing segregation methods to eliminate the *nptII* selectable marker gene. The resulting transgenic lines demonstrated high resistance to Colorado potato beetle, achieving up to 100% larval mortality in laboratory and field tests, with minimal damage to the plants [167].

Biotechnology methods, such as genetically modified crops expressing Bt toxins, proteinase inhibitors, and RNA interference, offer promising strategies to combat the Colorado potato beetle. However, the beetle’s extraordinary adaptive capabilities, including resistance development and compensatory mechanisms, necessitate the integration of these approaches into sustainable, multi-faceted pest management programs to ensure long-term effectiveness [168].

The genome of the cultivated potato *S. tuberosum* is allotetraploid and highly complex, which poses challenges for traditional crossbreeding methods [169]. Emerging NBTs like CRISPR/Cas systems, ZFNs, and TALENs have been utilized to achieve targeted mutagenesis and incorporate desirable traits, thereby improving agricultural productivity and resistance to biotic and abiotic stresses into elite cultivars [170]. Foreign DNA-free genome editing has been shown to be effective in species where protoplast cultures can be used with ribonucleoprotein complexes, such as apple, grapevine, and potato [171,172,173,174,175]. These advancements in genome editing and sequencing have revolutionized potato breeding, enabling the development of superior crop cultivars with enhanced nutritional profiles and higher yields [176].

Common potato (*S. tuberosum*) varieties are vegetatively propagated to maintain their characteristics, but they are also fully sexually compatible with other varieties and other *Solanum* species through advanced tissue culture techniques. Although potato breeding involves cross-hybridization, the resulting progeny may exhibit a very wide range of phenotypes, mostly undesirable due to pairing of homologous chromosomes and meiotic crossovers. In general, any crossover in an autotetraploid organism creates a huge number of combinations. Each of the four homologous chromosomes can pair with any other homologous chromosome during meiosis. Potatoes are a vegetatively propagated crop in agricultural environments and therefore they are genetically stable [177].

One of the applications of GM potatoes is their use in the development of edible vaccines. Potato-based edible vaccines have been developed to address the challenges of traditional vaccines, such as high costs and complex production processes, by leveraging the tuber’s ability to express transgenic proteins. Early studies demonstrated the successful expression of antigens like hepatitis B surface antigen, cholera toxin B subunit, and human papillomavirus virus-like particles in transgenic potatoes, showing their immunogenic potential in animal and human trials. Despite challenges like low protein yield and degradation during cooking, potato-based vaccines have shown promise in eliciting immune responses and are being explored for diseases such as dengue, cervical cancer, hepatitis B, diarrhea, cholera, and others [178].

Modern breeding strategies for late blight resistance in potatoes focus on identifying and introducing resistance genes from wild relatives or cultivated varieties using advanced genetic tools like marker-assisted selection, genome-wide association studies, effectoromics, and gene editing technologies such as CRISPR-Cas9. Additional approaches include gene pyramiding to stack multiple R genes for durable resistance, somatic hybridization to overcome natural hybridization barriers, and RNA interference or Host-Induced Gene Silencing to target pathogen susceptibility genes and enhance resistance [162].

#### 3.3.2. Gene Flow Through Pollen, True Seeds, and Volunteers

Gene flow through pollen and/or true seeds for potatoes has been estimated to be low. In Europe, there are no compatible wild plants for hybridization [179]. Many modern potato varieties are sterile, reducing seed sets. Potato flowers lack nectar, attracting only a limited range of pollinators like bumblebees, which are not widely distributed. Farmers typically do not collect potato berries, and their remains are eliminated during harvest [180].

In general, a 20 m distance has been determined to be sufficient for transgene containment in potatoes. This recommendation for field tests of transgenic potatoes is based on studies investigating pollen-mediated gene flow, including one of the earliest field studies conducted in the 1990s using transgenic potato (*S. tuberosum*) plants carrying the kanamycin resistance gene (*nptII*) [169]. In that study, non-transgenic potatoes grown side by side with transgenic rows exhibited a transgenic progeny frequency of 24%; this frequency declined to 2% at a distance of 3 m, to 0.017% at 10 m, and no gene transfer was observed at 20 m [169]. More broadly, studies assessing gene flow reported that the frequency of transgenic progeny in non-transgenic potatoes adjacent to transgenic plots ranged from 1% to 24% but declined rapidly to negligible levels within 3–5 m. Further comprehensive investigations confirmed that transgenic progeny frequencies dropped significantly at distances beyond 2.25 m, and isolation distances of 20 m were deemed sufficient to contain novel gene constructs during initial field tests [162]. Collectively, these findings support the recommendation of a 20 m isolation distance to ensure effective containment of transgenes and minimize the risk of gene flow to neighboring crops or related species [162,169].

Transgenesis and NGT present an accelerated way towards genetic improvement of potato, especially with new traits. However, it also brings an additional challenge to avoid foreign DNA sequences in the genomes of engineered potatoes, since the selfing and backcrossing would result in a high frequency of non-standard plants. Some scientists have highlighted that biotech potatoes pose minimal risk of gene flow to weedy relatives or conventional crops due to sexual incompatibility and limited pollen movement, ensuring that introduced traits remain contained. Additionally, biotech potatoes undergo extensive testing and regulatory review to ensure safety, with genetic modifications often being more precise and limited compared to traditional breeding methods [181].

For potatoes, both volunteer tubers left in the field and seeds that can germinate and create seedlings and tubers in the next vegetative season are considered as possible routes for environmental exposure of transgenes [36,60,182]. It has been estimated that 142,000–300,000 tubers per ha are left in the field, which is a higher number than the planting density for potatoes [60,183]. Tubers usually sprout in the next season, but the true seeds can remain dormant for two or more years [184]. A study conducted over a 10-year period in the UK showed that volunteers from transgenic potatoes were less common than those from traditionally bred varieties. For perennating potatoes, survival was significantly lower in GM lines in one of eight cases and was never significantly higher [185]. EFSA’s GMO panel considers the potato volunteer more as an agricultural problem but not as an environmental issue [185,186].

At the end of the 1990s, UK scientists concluded that GM potatoes can lead to contamination through pollen or seed dispersal, but the risk is reduced due to the partial male sterility of many potato varieties. They emphasized that contamination declines rapidly within short distances and is detectable up to 80 m, highlighting the importance of isolation measures to minimize genetic mixing [80]. At least one study indicated that a very low frequency of pollen tube formation is still possible for the male sterile lines. For example, five pollen tubes were observed out of 1110 grains in the b32RIP lines and one pollen tube out of 150 grains in the barnase lines, suggesting that while the frequency is significantly reduced, it is not entirely eliminated [187].

In the early 2000s, it was evaluated that field trials with insect-resistant transgenic potatoes and other genetically modified crops have not revealed any indications of enhanced competitiveness or invasiveness outside agricultural systems compared to their non-transgenic counterparts. Transgenic potatoes have shown similar ecological behaviour, with no evidence of sexual reproduction in natural ecosystems, suggesting limited environmental impact [188]. Two experiments were carried out with weed species *Solanum dulcamara* L. and *Solanum nigrum* L. growing close to transgenic potatoes. Viable offspring and gene flow to these wild species were not observed [189,190]. Other researchers have concluded that there are no wild species related to the potato in the main potato-producing countries [191].

In the South American regions where potatoes originated, many wild *Solanum* species exist. Hybrid development was analyzed under field conditions by forced and open pollination among the *S. tuberosum* cultivar group Andigenum and six wild species: *Solanum albicans* (Ochoa) Ochoa, *Solanum acaule* Bitter, *Solanum chomathophilum* Bitter, *Solanum raphanifolium* Cárdenas & Hawkes, *Solanum bukasovii* Juz. ex Rybin, and *Solanum sparsipilum* (Bitter) Juz. & Bukasov. By open pollination, only three wild relatives crossed with *S. tuberosum*. However, in the study, the authors emphasized the need for precautionary measures, such as using male-sterile cultivars, to prevent gene flow to wild relatives, while further evaluating the long-term ecological and evolutionary risks of transgene introgression in the Andes, a center of potato biodiversity. [192]. In another study in Peru, scientists crossed wild *Solanum candolleanum* Berthault and 30 cultivated potato varieties. Seed production was detected in almost 35% of the crosses [193]. In a literature study, it was evaluated that in Latin America, there could be some risks regarding GM herbicide-resistant potato varieties and wild relatives. The authors highlight that while hybridization between GM herbicide-resistant potato varieties and their wild relatives is limited due to pre- or postzygotic barriers, there are instances where interspecific crosses can occur without sterility barriers, particularly in centers of diversity like Mexico and the Andes. They emphasize the need for precautions to prevent transgene introgression into native populations, as hybrids with enhanced fitness could pose ecological risks, although existing studies suggest minimal risk in certain regions like Costa Rica [194]. If GM potatoes were introduced, their genes could flow into wild relatives and native cultivars, potentially impacting genetic diversity and biosafety. Cultivation of male-sterile varieties, such as ‘Revolución’, could minimize this risk [195].

A methodology was developed to assess the outcrossing potential of 11 crop species in Chile with wild relatives or introduced relatives, but no practical field trials were conducted. The highest number of native and introduced relatives were potatoes and tomatoes. The outcrossing potential in various parts of the country for potatoes was evaluated as medium-high [196]. In a study in Argentina, transgenic potato virus Y-resistant potatoes *S. tuberosum* ssp. *tuberosum* (cv. Spunta) were grown in close proximity to the wild relative *Solanum chacoense* Bitter. Among 41,000 seeds of *S. chacoense* tested, none of the 100 pooled seed groups contained the transgenic sequence [197]. Similarly, no gene flow was detected in a study in the Peruvian Andes, where non-GM commercial variety Yungay (donor plant) was grown by local farmers for 15–25 years, along with more than 400 landraces (acceptor plants) [198]. However, field evidence of pollen-mediated gene flow exists. Experimental field work showed pollen-mediated transfer from a cultivated potato to a wild relative (e.g., *S. chacoense*) under experimental conditions up to 30 m from the pollen donor [199]. The authors of the previously mentioned review state that the potential genetic flow between GM potatoes and wild relatives is a concern, particularly in the Andean region, the center of origin for potatoes. However, they note that intercrossing between cultivated potatoes and wild relatives is generally restricted by reproductive barriers, and field trials in Argentina have shown no transgene transfer under natural conditions [194]. To explain the different results obtained in the above-mentioned studies, it is probably necessary to consider the similarity and differentiation of the genomes of wild potato and cultivated potato, as well as the different ploidy combinations [200].

Scientists in Indonesia conducted a gene flow study to determine the potential for gene transfer from GM potatoes to non-GM potatoes in the field. The study revealed that gene flow occurred through natural hybridization at varying isolation distances, significantly decreasing with increasing isolation distance: at 0.8–1.6 m, the gene flow rate was 13.78%; at 2.4–4 m, the rate decreased to 10.92%; at 4.8–6.4 m, the rate further decreased to 3.82%, at a distance of 7.2–8.0 m, the gene flow rate declined to 0%. This suggests that maintaining sufficient isolation distances can mitigate the risk of gene transfer from GM potatoes to non-GM varieties [201].

Since the largest part of commercial potato varieties are sterile, more possible routes for gene flow from transgenic potatoes can occur via potato tuber volunteer plants and overwintered groundkeepers (tubers left in the soil after harvest) that can develop into feral potato plants [202]. Nevertheless, some potato varieties do shed pollen, like the conventional variety Désirée. In a field study in Ireland, berries were formed on receptor variety plants up to 21 m away, but only a small amount of the seeds were viable. Irish co-existence guidelines for GM potato cultivation recommend a 20 m isolation distance to limit transgenic pollen spread. The study extended the distance slightly further than 20 m to evaluate whether gene flow could occur beyond the recommended threshold under Irish environmental conditions [203]. Non-flowering genotypes could be the most suitable option for gene containment in transgenic potatoes as well as transgene elimination during pollen development [184].

Finnish scientists highlighted that volunteer GM potatoes pose minimal risk to the coexistence of GM and conventional potato production in northern Europe due to cold winter conditions that typically kill most tubers left in the soil after harvest. However, seedlings from true potato seeds can survive and produce tubers despite the short growing season, presenting a potential risk that could be mitigated by cultivating non-berry-producing GM potato varieties [204]. Some scientists noted that higher winter temperatures expected under future climate scenarios could increase the survival of groundkeeper potatoes, making volunteer control an ongoing concern for farmers and GM field experimenters [60,205]. Some scientists discussed the issue of gene flow and the potato volunteer problem in the context of managing GM potatoes. They highlighted the importance of controlling potato volunteers to minimize the risk of GM contamination in non-GM potato crops. To address this, the authors proposed best management practices (BMPs) in their identity preservation systems, including: rotate no more than one of four years in potatoes; follow GM potatoes with crops likely to reduce volunteers or with summer fallow; and apply maleic hydrazide during the GM potato growing season to reduce volunteer growth. These practices aim to mitigate the risk of gene flow and contamination by controlling the growth of volunteer GM potatoes in subsequent planting seasons [206].

In a recent article by Norwegian scientists, the authors argued that GM potatoes pose minimal risk of gene escape to wild relatives in Western Europe, as *S. tuberosum* is not sexually compatible with common species like black nightshade (*S. nigrum*) and bittersweet (*S. dulcamara*). They also suggested that implementing regulations on physical distance, growing intervals, and machinery disinfection can further reduce the risk of gene spread and potential ecosystem effects [207].

The predicted distances of gene flow for various potato species are given in Table 7.

#### 3.3.3. Other GM Crops from the *Solanum* Genus

Only a few other members of the *Solanum* genus have been genetically modified. One example is eggplant *Solanum melongena* L.: Bt brinjal Event EE-1. There is limited but relevant information available on the potential for gene flow and hybridization between the Bt brinjal event EE-1 and non-GM plants or wild relatives. A study conducted in southern India assessed the potential for crop-to-wild gene flow between cultivated brinjal and its wild relative, *Solanum insanum* L. The findings indicated that hybridization is possible, as hand crosses between wild and cultivated brinjal resulted in viable F1 progeny. The presence of effective pollinators such as bees, which forage on both wild and cultivated brinjal, further supported the likelihood of gene flow. This suggests that transgenes from Bt brinjal could potentially spread to nearby wild brinjal populations [210].

In a recent review article, authors highlighted that transgenic and cisgenic techniques are powerful tools for rapidly transferring beneficial traits from crop wild relatives to cultivated eggplants, potatoes, and tomatoes, enabling enhanced resilience, productivity, and sustainability. However, they noted that the application of these methods is limited due to regulatory restrictions in many countries [209].

Targeted gene modifications with the CRISPR/Cas system have been adapted for potatoes. CRISPR/Cas systems can be constitutive and/or viral-based using AMT methods [211]. AMT leads to the insertion of a transgene into the genome, and in the case of potatoes, it cannot be segregated away by crossing. Instead, protoplast transformation with ribonucleoprotein (RNP) can be used [171]. Other scientists suggest using genome editing technologies, such as CRISPR−Cas9, to precisely modify potato genes without integrating foreign DNA, regarding biosafety concerns and regulatory challenges. Protoplast transfection with ribonucleoproteins (RNPs) can achieve high numbers of edited plants while avoiding foreign DNA integration [208]. Genome editing technologies can be used in potato breeding to improve nutritional quality, modify tuber starch composition, enhance post-harvest quality, increase resistance to biotic stress, and address reproductive self-incompatibility issues [212]. 

#### 3.3.4. Conclusions on Potato

The overall conclusions are that there is very little experimental data on hybridization between GM potatoes and wild relatives or the potato volunteer problem in the last 10 years. Potato is vegetatively propagated, which reduces opportunities for widespread hybridization. Most cultivated potatoes (*S. tuberosum*) are tetraploid, often with poor flowering and male sterility, limiting natural hybridization. Experimental studies from the 1990s showed that pollen-mediated gene flow is possible only at very short distances (up to ~20 m), with rapid decline beyond that. Crosses with wild relatives in South America are possible but vary depending on species and ploidy, and systematic field surveys are lacking. European studies show little risk due to a lack of compatible wild relatives.

However, volunteer tubers and true seeds can survive in soil, providing possible routes for persistence. Estimates of tuber persistence exist, but the ecological significance of volunteers and long-term population establishment is poorly studied. In the future, systematic studies of tuber persistence, overwintering, and volunteer populations across climates are needed.

In general, there is limited information on whether hybrids with wild *Solanum* would have selective advantages or enhanced persistence.

Most of the experimental data originate from studies over 20 years old and do not include potatoes engineered by NGTs. Existing information includes mostly review articles, opinion pieces, or articles about the future potential of the new breeding methods. Potato genetic engineering efforts often appear in the context of plant-based vaccines and other similar applications. Nevertheless, there is a number of indications that NGT potatoes are under development, and field trials are underway in the EU [171,213].

Although hybridisation with wild relatives is not a concern in Europe, further research on GM potatoes is essential because traits such as late blight (*Phytophthora infestans* (Mont) de Bary) resistance and drought tolerance could alter the persistence and management of volunteer plants. Potato remains highly vulnerable to *P. infestans*, causing global losses and reliance on fungicides [162]. GM approaches show potential to reduce pesticide inputs and improve yield stability, but require rigorous evaluation of ecological effects, soil interactions, and food safety [53]. Moreover, climate change increases abiotic stress risks, making genetic innovations vital for long-term food security [214]. Thus, research is needed not because of gene flow risk in Europe but to ensure that GM potatoes deliver sustainable, safe, and resilient production.

### 3.4. Analyses of Search Results on NGT Plants

EFSA has been tasked by the EC with regular horizon scanning to assess new scientific data on plants, animals, microorganisms, and products thereof obtained by NGTs, to assess any new data and evidence emerging from these studies, and to consider whether it may have implications for EFSA’s relevant scientific opinions [215]. The first horizon scan focused on plants and their products obtained by NGTs. The horizon scan identified six scientific studies as relevant after full text screening; however, they were assessed not to have any implications for the previous EFSA opinions on the safety of NGT plants, and none of them concerned oilseed rape or potato [215]. In 2026, EFSA issued the second horizon scan looking at plants, microorganisms and animals and their products obtained by NGTs with the same conclusion of no implications on the conclusions of the previous relevant EFSA opinions [216].

While the horizon scan did not focus specifically on gene flow, it aimed at identifying novel hazards caused by genome editing, which would change the EFSA’s previous opinion on plants created by site-directed nucleases (SDNs) SDN-1 and SDN-2 [217,218] and the updated opinion on cisgenesis and intragenesis [11]. The horizon scans concluded that the previous EFSA conclusions on NGT plants and their products do not present any new hazards compared to conventionally bred plants, and remain unchanged, including ERA.

The largest number of studies on the biosafety of NGT plants can be found on rice. One potential issue regarding rice could be the transfer of herbicide resistance, like ALS-resistance (acetolactate synthase resistance), to related wild species, regardless of the method (GM or NGT) used to obtain this trait for the cultivated variety [56]. Rice, in general, has been modified the most often with CRISPR/Cas in comparison to other crop plants [219].

In recent years, potatoes have emerged as the crop with the most field trials (EC GMO register, Part B notifications) in European countries, both for GM potatoes and NGT potatoes (https://ec.europa.eu/food/food-feed-portal/screen/gmob/search, accessed on 17 March 2026). Six field trials of potatoes are ongoing in Denmark and Sweden, of which one trial involves a cisgenic potato and three trials involve genome-edited (presumably NGT1) potatoes. Oilseed rape is not among the top 10 crops tested in field conditions. Among oilseeds, field trials are conducted with camelina [220]. The EFSA’s GMO Panel concluded that site-directed nucleases SDN-1, SDN-2, and ODM approaches do not pose any additional hazards compared to SDN-3 and traditional breeding techniques, similar to conventional mutagenesis. Although the SDN-1 and SDN-2 methods may cause off-target genetic changes, the GMO panel noted that these are less common than changes resulting from classical random mutagenesis, which reduces the potential for gene alteration or interruption [217]. Persistence and invasiveness have been considered as some of the main risks of future genome-edited plants. Subtle genomic modifications leading to changes in flowering time, plant architecture, or reproductive traits can enhance the likelihood of hybridization with wild relatives or other plants. For example, *de novo* domesticated plants like wild tomatoes (*Solanum pimpinellifolium* L.) may retain traits that increase their compatibility with wild species, leading to gene flow and the spread of modified traits in natural ecosystems. Genomic changes that improve stress tolerance, competitive ability, or seed survival can increase the persistence and invasiveness of hybrids [221,222]. This is particularly concerning for non-familiar plants or weeds modified through NGTs, as their environmental behavior is less predictable [14]. Other main concerns regarding transgene flow are the potential ecological and biosafety risks, such as the evolution of aggressive weeds, extinction of wild relatives, genetic erosion, and biodiversity loss due to gene introgression. Additionally, horizontal gene transfer to unrelated organisms and unintended effects on non-target species, including pollinators, raise significant environmental and health concerns [76], although these do not appear to be uniquely associated with NGTs. Even though CRISPR/Cas has been heralded as a paradigm-changing technology in plant breeding [223], the challenges for applications related to AMT dependency and low efficiency of editing still remain [224,225]. Additionally, inconsistent regulatory policies and the lack of clear policies on field trials to evaluate the final performance of CRISPR-edited crops hinder their commercial production and adoption [226]. The use of CRISPR-Cas9 and similar SDNs has been assessed by the EFSA, which concluded that the use of SDN-1 and SDN-2 approaches does not create any additional hazards compared with transgenesis and conventional breeding [217]. This scientific opinion, along with a number of other opinions and studies, was used by the EC to come up with a proposal for regulating NGTs [13].

CRISPR/Cas9 has been used in precision breeding of oilseed rape, with multiple demonstrated uses, including reduced shatter resistance of pods, reduced erucic acid content, downregulated aliphatic glucosinolate biosynthesis genes, and many others (reviewed by Boniecka J (2024) [227] and Muhtiar et al. (2025) [228]).

In potato, a recent review by Vidya and Arun (2023) [229] provides an update on the development of genome editing techniques and emphasizes its use for yield enhancement, improved stress tolerance, and nutritional enhancement. Notably, genome editing in potato can generate non-transgenic plants through DNA-free editing to develop starch with an increased amylose ratio and elongated amylopectin chains with a higher glycemic index after food intake and better properties for bioplastic films [171]. In another review, a GM potato modified with the *P5Cs* gene from *Arabidopsis thaliana* (L.) Heynh. was highlighted, which increases salt tolerance and proline content while maintaining tuber yield and weight. The authors highlighted general concerns about environmental risks associated with genetically modified crops, including techniques like CRISPR-Cas9, such as unintended gene flow to wild relatives and the potential for hybridization, which could lead to invasiveness. They emphasized the need for comprehensive risk assessments and regulatory measures to address these ecological impacts responsibly [230].

Other scientists have argued that concerns about gene transfer from GM crops to non-target organisms are unfounded, as extensive evaluations over 28 years have not identified any harm to consumers or the environment, including DNA transfer. They emphasize that GM crops undergo rigorous testing and are as safe or safer than conventional crops, but precautionary assessments should continue to address potential future concerns [231]. The use of NGTs for genome editing in plants is currently in its infancy, with only a limited number of products on the market, which were created by the simplest possible techniques, such as simple gene knockouts or single-nucleotide substitutions. Since genome editing can mirror natural processes in plant genomes, it may, in some cases, be reasonable to use the full potential of natural genetic processes accelerated by NGTs [232].

Biosafety concerns in conventional transgenic crops were raised because they involve the introduction of foreign DNA, which may present environmental and food safety risks [233]. To mitigate these issues, they emphasize cisgenic and intragenic strategies—using genetic material from the same or closely related species—as well as genome-editing technologies such as CRISPR/Cas9, which enable precise genetic modifications without incorporating foreign DNA, thereby improving environmental compatibility and potential consumer acceptance. As examples, they discuss genetically modified canola with altered oil composition, including omega-3-enriched varieties produced by introducing a fungal Δ-6 desaturase gene, and several potato innovations: transgenic potatoes expressing the AmA1 seed albumin to enhance yield and protein quality, cisgenic potatoes resistant to late blight caused by Phytophthora infestans, and intragenic potatoes engineered to reduce acrylamide formation during French fry processing [233].

In a recent review, authors stated that cisgenic crops pose similar environmental risks as traditionally bred plants, while genome-edited crops can be designed to avoid foreign gene insertion, making them genetically similar to non-transgenic crops and potentially less concerning for environmental safety. However, genome-edited crops involving transgene insertion may still be subject to the same stringent regulatory processes as transgenic crops due to potential environmental risks [234]. In another review, authors emphasized that marker-free transgenic plants are environmentally safer than first-generation transgenic crops as they eliminate the risk of horizontal gene flow of antibiotic or herbicide resistance genes to non-target organisms, which could lead to ecological imbalances. They highlight that the absence of marker genes in transgenic plants reduces potential damage to ecosystems and addresses regulatory and consumer concerns about the safety of genetically modified crops [235].

Development of a regulatory system that would allow for the use of the full potential of NGTs is also needed, especially in the EU, where NGT plants are still regarded as GMOs [5]. While the EC legislative proposal is a step in the right direction, it is only one of the regulatory options available [236] and represents a very conservative approach, with many compromises that do not fit the established facts about natural genetic variation in plant genomes [16]. In conclusion, vertical transgene flow is a well-established phenomenon. Its potential environmental implications depend on the crop species and ecological context and are evaluated during the authorization process. There is little, if any, data on the potential gene flow from NGT plants and its environmental effects. It can be speculated that conventional-like NGT1 plants, which would have the same environmental impact as the conventionally bred plants with the same potential for increased or decreased fitness, would be assessed through the usual variety testing and registration process. NGT2 plants would still require authorization—at least in the EU—triggering the standard ERA process in which the potential for gene flow would be assessed; however, gene flow itself is not a risk but rather a frequency or exposure component within the risk formula, contributing to the overall quantification of risk.

## 4. Conclusions

**Figure 1:**
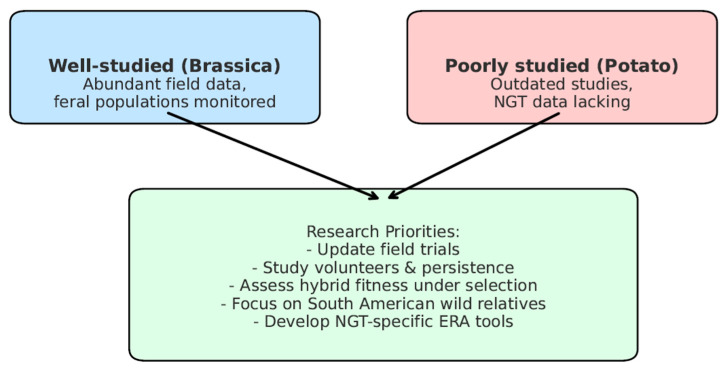
Conceptual synthesis of research gaps identified throughout the literature discussed in the manuscript (created with ChatGPT-5, https://openai.com/ (accessed date 15 September 2025)). The figure summarizes differences in the current knowledge base between *Brassica napus* L. (extensively studied, including field evidence and feral population monitoring) and potato *Solanum tuberosum* L. (limited and often outdated field studies and little NGT-specific information) and highlights key areas where further research would support environmental risk assessment. Research priorities include updated field trials, studies on volunteer tuber persistence and seed bank ecology, evaluation of hybrid fitness under selective pressure, targeted research in South American centers of origin, and development of NGT-specific environmental risk assessment tools.

The overall conclusions acknowledge that gene flow from genetically modified (GM) crops can present potential ecological risks; however, the magnitude and nature of these risks differ substantially among species. This review emphasized that empirical data on hybridization between GM plants and their wild relatives remain limited, particularly in the case of potatoes, where most available research has concentrated on other crops (Figure 1). While additional studies on gene flow in potatoes may offer only limited new insights—except perhaps in South America, where compatible wild relatives are present—further research into the environmental implications of new genomic techniques (NGTs) remains essential. NGTs hold the promise to deliver more diverse traits, which may affect plant fitness and invasiveness, although the current assumption is that in some cases these plants will remain conventional–like. In Europe, future investigations should focus on understanding the persistence, dispersal, and ecological behavior of GM and NGT potatoes possessing novel traits. Moreover, given the unpredictability associated with altered phenotypes or enhanced fitness in oilseed rape and other *Brassica* species, such varieties should be subject to rigorous, case-by-case biosafety assessments. Overall, sustained research on the interactions between GM plants and their wild relatives is crucial to ensure informed, scientifically sound evaluations of environmental risk and biosafety.

From a methodological point of view, the authors conclude that the number of publications regarding GM plants is large, demanding extensive resources for in-depth literature analysis, including time, funding, and specialized literature analysis software, as well as artificial intelligence tools.

## Figures and Tables

**Table 1:** Factors that determine the likelihood of a hybrid between a crop in general and a related species establishing itself in an agricultural or natural habitat [32].

No.	Factor
Formation of viable hybrid seed	
1	Compatibility of the genomes of both parents (mitotic and genetic stability)
2	Endosperm’s ability to support hybrid embryo development
3	Crossover direction: one parent can support embryo and seed formation better than the other
4	Number and viability of hybrid seeds
Establishing hybrid plants in the soil	
5	Seed dormancy
6	Hybrid plant vitality
7	Direction of crossing: influence of the mother plant on seed vitality
8	Habitat characteristics: wild, semi-wild, or agricultural
9	Peculiarities of competition with other plants
10	Impact of pests, diseases, and herbivores
A hybrid’s ability to reproduce vegetatively and sexually	
11	Vegetative propagation method
12	Preservation of vegetative plant parts in agricultural environments
13	Vegetative propagation
14	Invasiveness of vegetative parts in natural habitats
15	Sexual reproduction system: cross-compatibility, self-pollination, ability to cross with any of the parent species
16	Male and female fertility: meiotic stability and chromosome conformity
17	Seed number and viability
18	Seed dormancy
19	Habitat characteristics: wild, semi-wild, or agricultural
20	Peculiarities of competition with other plants
21	Impact of pests, diseases, and herbivores

**Table 2:** Pre- and post-hybridization transgene management strategies (summarized by Kwit et al., 2011) [50].

Problem	Strategy
Pre-hybridization	
Pollen formation	Genetic male sterility
Paternal inheritance	Cytoplasmic male sterility
Synchronous flowering	Delayed flowering
Pollen-containing transgene	Transgene excision in pollen
Open flower	Cleistogamy
Post-hybridization	
Equal chance of introgression	Linkage disequilibrium
Compatible ploidy level	Ploidy barrier
Competitive weeds	Transgene mitigation
Competitive weeds	Selectively terminable transgenic lines *

* Selectively terminable transgenic lines are engineered for targeted removal. In rice, an RNAi cassette suppresses the bentazon detoxification gene (*CYP81A6*), making transgenic plants sensitive to bentazon and allowing their selective elimination, while leaving non-transgenic plants unaffected [50].

**Table 3:** Comparative summary of literature search results (oilseed rape vs. potato).

Crop/Focus	Total Hits (Range)	Relevant % (Approx.)
Oilseed rape/*Brassica* spp.	100–200+ (WoS, PubMed *)	30–100% depending on the search string
Potato	Few to hundreds (varied by database)	0–43% depending on the search string

* WoS—Web of Science database, PubMed—free online database of biomedical and life sciences literature, maintained by the USA National Library of Medicine at the National Institutes of Health.

**Table 4:** Observed gene flow distances in *Brassica* systems (created with Acrobat AI Assistant).

Donor → Recipient	Key Context	Cross-Pollination at Set Distances	Farthest Distance with Detected Gene Flow	Source
*B. napus* → *B. napus* (crop to crop)	Field plots with bar transgene; mixed pollination (wind + insects)	~4.8% when plants in close contact; 1.5% at 1 m; 0.00033% at 47 m	Rare, very low levels detected up to several km in landscape settings	[22,77]
*B. napus* → *B. napus* (crop to crop; seed standards)	Isolation distance tests for small plots	0.156% at 200 m; 0.0038% at 400 m	-	[78]
*B. napus* ↔ *B. napus* (commercial field scale)	Adjacent 10 ha blocks; transects into conventional field	Mean cross-pollination declines from edge; kept <~0.5% by ~100 m in fully fertile crops (site-dependent)	Low levels occasionally extend to kilometers; pollen recorded to several km	[22]
*B. napus* → *B. napus* (landscape)	Commercial canola fields	Rare pollen-mediated movement observed between fields of up to ~3 km	~3 km (rare events)	[79]
*Brassica* (general) → *Brassica*	Mixed studies summarized	Example contamination reports: 0.4% at 12 m, 6% at 137 m (context-dependent)	Pollen detected to ~1.5 km; low frequencies at long range	[80]
*B. napus* → *B. rapa* (crop/weedy)	Field proximity, sympatry	Hybridization common when co-occurring; within/adjacent fields, 1–17% hybrids in *B. rapa* reported (rates vary with distance and relative abundance)	Gene flow documented up to ~200 m in crop settings	[74,75]
*B. napus* → *B. juncea*	Adjacent fields in production regions	Very low frequency: 0.024% hybrids detected	Up to 400 m	[76]
*B. napus* → *B. carinata*	Adjacent fields	Very low frequency: 0.005% hybrids detected	Up to 150 m	[76]
*B. napus* → *Raphanus raphanistrum* (wild radish)	Normal agronomy; wild radish placed in and around fields	Interspecific hybrids detected at very low frequencies (e.g., ~10^−6^–10^−5^ in large field trials); highest at field border	Gene flow concentrated at field edge; long-range not quantified	[20]
*B. napus* → *B. oleracea* (wild cabbage)	Natural populations (coasts); molecular detection	Spontaneous gene flow detected; rates context-specific, typically low	Not specified	[75]

**Table 5:** Documented cases and potential occurrence and persistence of transgenic oilseed rape populations in various ruderal habitats across multiple countries (generated with the help of Scopus AI).

Country	Described Issue	Reference
Canada	Transgenic herbicide-resistant oilseed rape was documented in ruderal areas such as railways and roads in Saskatchewan and at the port of Vancouver, British Columbia	[138,139]
China	Research indicates the presence and environmental risks of transgenic oilseed rape, with a focus on gene dispersal and biodiversity impact	[140]
Japan	Transgenic oilseed rape plants were found at several ports, roadsides, and riverbanks in the Kanto District and other regions	[109,141,142]
Switzerland	Surveys detected genetically engineered glyphosate-tolerant oilseed rape in railway areas, including Lugano and Basel	[143]
Germany	Although the cultivation of transgenic oilseed rape is banned, studies were conducted to assess the potential spread and persistence of these plants	[127,144]
Austria	Feral populations of non-modified oilseed rape were studied for genetic diversity and persistence, indicating potential hybridization with transgenic varieties	[145]
France	Genetic relationships among oilseed rape genotypes from France were evaluated, suggesting the presence of transgenic varieties from former cultivation	[63,146]
Australia	Transgenic oilseed rape outside cultivation area was detected in Western Australia	[147]
United Kingdom	Predictions indicate the potential for second-generation transgenic hybrids in natural populations	[148]
Argentina	Transgenic glyphosate-resistant oilseed rape was detected as an invasive weed in the fields of other crops	[149,150]

**Table 6:** The key factors influencing pollen-mediated gene flow at the landscape scale [151] (generated with the help of Acrobat AI Assitant).

Category	Factors
Plant Characteristics	- Plants have to be sympatric - Reproductive capacity
	- Breeding system (self-pollinating vs. cross-pollinating)
	- Flowering synchronization, pollen viability, compatibility
	- Pollen dispersal patterns
Field and Landscape Factors	- Field size
	- Planting structure
	- Proximity of populations
Environmental Conditions	- Geographical and meteorological factors (e.g., wind speed, direction, climate)
	- Landscape structure (connectivity and fragmentation)
Human Activities	- Agricultural practices (e.g., seed sharing, saving, transportation)
	- Trade and transport of seeds and crops
Biological Interactions	- Role of pollinators (e.g., bees, flies)
Crop-Specific Factors	- Genotype and transgene traits (e.g., herbicide or insect resistance)
	- Seed dormancy and persistence

**Table 7:** The predicted distances of gene flow for various potato (*Solanum*) species (generated with the help of Adobe AI).

Donor → Recipient	Context	Cross-Pollination at Set Distances	Farthest Distance with Detected Gene Flow	Source
*S. tuberosum* → *S. tuberosum* (crop to crop)	Field trials with GM potatoes in Europe	24% when plants are adjacent; 2% at 3 m; 0.017% at 10 m; 0% at 20 m	20 m 80 m	[22,76,80,189]
*S. tuberosum* → *S. tuberosum* (crop to crop)	Field trials with GM potatoes in Indonesia	13.78% at 0.8–1.6 m; 10.92% at 2.4–4 m; 3.82% at 4.8–6.4 m, 0% at 7.2–8.0	6.4 m	[201]
*S. tuberosum* → *S. chacoense* (wild relative)	Experimental field conditions in Argentina	Hybrid seeds detected at 30 m	30 m	[199]
*S. tuberosum* → *S. nigrum* (wild relative)	Field trials in Europe	No evidence of gene flow at 20 m	20 m	[22,189]
*S. tuberosum* → *S. dulcamara* (wild relative)	Field trials in Europe	No evidence of gene flow at 20 m	20 m	[22,189]
*S. tuberosum* → Wild *Solanum* species (general)	Field trials in Peru	Gene flow detected within 3 m; hybridization confirmed in field trials	3 m	[192,195]
*S. tuberosum* → Wild *Solanum* species (general)	Field trials in the Andes	Gene flow detected within 3 m; hybridization confirmed in field trials	3 m	[208,209]

## Data Availability

No new data were created or analyzed in this study.

## Supplementary Materials

The following supporting information can be downloaded at https://www.mdpi.com/article/10.3390/biotech15020030/s1. Table S1. Results of preliminary searches in several databases by combining variations of the key elements of the review question Q1 with AND (searched in September 2024–July 2025). Table S2. Most frequently described hybridization cases by plant species, family etc. in the literature according to the search term No. 1 “Genetically modified plants AND hybrid AND wild plants” in the Web of Science database (n = 129). Table S3. Results of preliminary searches in several databases by combining variations of the key elements of the review question Q2 with AND (searched in September 2024). Table S4. More elaborated search string variants for hybridization in general and for potatoes and canola in particular (searched in August 2025). Table S5. Search strings for NGTs elaborated from Eckerstorfer et al. (2019) [56] (searched in June–August 2025). Table S6. Search strings in PubMed and Europe PMC using controlled vocabulary thesaurus of Medical Subject Headings (MeSH). Figure S1. PRISMA flow diagram for the searches of databases.

## Abbreviations

The following abbreviations are used in this manuscript:

ALS	Acetolactate synthase
AMT	*Agrobacterium*-mediated transformation
Bt	*Bacillus thuringiensis* toxin-producing
Cas	CRISPR-associated
CRISPR	Clustered regularly interspaced short palindromic repeats
EC	European Commission
EFSA	European Food Safety Authority
ERA	Environmental Risk Assessment
EU	European Union
Europe PMC	Europe PubMed Central
GE	Genetically engineered
GenAI	Generative artificial intelligence
GM	Genetically modified
GMHT	Genetically modified herbicide-tolerant
GMO	Genetically modified organism
MeSH	Medical Subject Headings
NGT1	Category 1 NGT plants
NGT2	Category 2 NGT plants
NGTs	New genomic techniques
ODM	Oligonucleotide Directed Mutagenesis
OECD	Organisation for Economic Co-operation and Development
PMEM	Post-market environmental monitoring
PubMed	Free online database of biomedical and life sciences literature, maintained by the USA National Library of Medicine at the National Institutes of Health
RA	Risk assessment
RNPs	Ribonucleoproteins
SDN	Site-directed nuclease
TALENs	Transcription Activator-Like Effector Nucleases
TM	Transgenic mitigation
UK	United Kingdom
USA	United States of America
WoS	Web of Science database
ZFN	Zinc Finger Nuclease
