# Supplementary material for: Gene Flow and Hybridization Potential Between GM/NGT Crops and Conventional Varieties or Wild Relatives: A Scoping Literature Review with Emphasis on Oilseed Rape (Brassica napus L.) and Potato (Solanum tuberosum L.)
Source: BioTech (Basel). 2026 Apr 8;15(2):30. doi: 10.3390/biotech15020030 (PMC13108222; doi:10.3390/biotech15020030)
Supplement: Supplementary file 1 [file biotech-15-00030-s001.zip › biotech-4065105-supplementary.pdf]

Supplementary material

Table S1. Results of preliminary searches in several databases by combining variations of the key elements of the review question Q1 with AND (searched in September 2024 – July 2025).

No.	Search string	Number and percentage of compliant entries in various databases (n, %)			
		**PubMed**	**Web of Science**	**Scopus**	**Europe PMC**
1	Genetically modified plants AND hybrid AND wild plants	1083 (NA)	177 (126, 71.19%)	9803 (NA)	7873 (NA)
2	Genetically modified plants AND hybrid AND conventional varieties	42 (5, 11.90 %)	27 (9, 33.33 %)	2822 (NA)	1408 (NA)
3	Genetically modified plants AND sexual crossing AND wild plants	12 (2, 16.67 %)	8 (3, 37.5 %)	373 (22 out of 70, 31.43 %)	980 (NA)
4	Genetically modified plants AND (hybrid OR gene flow) AND wild plants	1135 (NA)	297 (73 %)	6241 (NA)	8682 (NA)
5	Genetically edited plants AND hybrid AND wild plants	88 (0 %)	2 (2, 100 %)	797 (NA)	3259 (NA)

NA – not assessed.

Table S2. Most frequently described hybridization cases by plant species, family etc. in the literature according to the search term No. 1 "Genetically modified plants AND hybrid AND wild plants" in the Web of Science database (n=129).

Species, family etc.	Number of articles	Species, family etc.	Number of articles
Brassica, oilseed rape, *Brassica juncea, B. rapa, Brassica oleracea*	36	Sorghum (*Sorghum bicolor*)	2
Various species or general	14	Tobacco (*Nicotiana tabacum*)	2
Rice (*Oryza sativa*)	11	Tomato (*Solanum lycopersicum*)	2
Sugar beet, *Beta vulgaris*, *Beta maritima*	8	Charry (*Cerasus avium*)	1
Maize (*Zea mays*), teosinte (*Zea* spp.)	6	Chicory (*Cichorium intybus*)	1
Lettuce (*Lactuca sativa*)	5	Chili pepper (*Capsicum annuum*)	1
Soybean (*Glycine max*)	5	Creeping bentgrass (*Agrostis stolonifera*)	1
Sunflower (*Helianthus annuus*)	5	Foxtail millet (*Setaria italica*)	1
Squash (*Cucurbita* spp.,), eggplant (*Solanum melongena*), melon (*Cucumis melo*)	4	*Kalanchoe blossfeldiana*	1
Strawberries (*Fragaria* × *ananassa*)	4	*Medicago falcata/sativa*	1
Wheat (*Triticum aestivum*)	4	Poplars (*Populus* spp.)	1
Cotton (*Gossypium* spp.)	3	*Rosa*	1
Sugarcane (*Saccharum officinarum*)	3	Seaweeds	1
Camelina sativa	2	*Vigna angularis*	1
Carrot (*Daucus carota* subsp. *sativus*)	2	Potato (*Solanum tuberosum*)	0

Table S3. Results of preliminary searches in several databases by combining variations of the key elements of the review question Q2 with AND (searched in September 2024).

No.	Search string	Number and percentage of compliant entries in various databases (n, %)						
		PubMed	Web of Science	Scopus	Europe PMC	Google Scholar	Science Direct	Base
1	Genetically modified potato AND hybrid AND wild plant	30 (1, 3.33 %)	13 (5, 38.46 %)	2776 (NA)	1445 (NA)	46,500 (NA)	4149 (NA)	18 (0, 0 %)
2	Genetically modified potato AND hybrid AND conventional varieties	2 (1, 50 %)	0	1119 (NA)	507* (4 %)	33,500 (NA)	3676 (NA)	6 (0, 0 %)
3	Genetically modified potato AND sexual crossing AND wild plant	0	0	148 (20 %)	278** (22, 27.16 %)	19,800 (49 %)	626 (NA)	0
4	Genetically modified potato AND (hybrid OR gene flow) AND wild plant	36 (3, 8.33 %)	16 (7, 43.75 %)	1538 (NA)	1417 (NA)	47,800 (NA)	6151 (NA)	120 (NA)
5	Genetically edited potato AND (hybrid OR gene flow) AND wild plant	1 (0, 0 %)	1 (1, 100 %)	135 (37 %)	599* (0 %)	24,900 (NA)	2524 (NA)	72 (0, 0 %)
6	Genetically modified potato AND volunteer tuber AND field trial	0	2 (1, 50.00 %)	68 (8, 11.76 %)	36 (0 %)	15,300 *** (42 %)	232*** (13 %)	0

NA – not assessed. *- First 100 entries grouped chronologically starting from the oldest were detailly assessed. The results are obtained by extrapolation from the first 100 entries. **-81 was detailly assessed, other were screened only by title. ***- First 100 entries grouped by relevance were detailly assessed. The results are obtained by extrapolation from the first 100 entries.

Table S4. More elaborated search string variants for hybridization in general and for potatoes and canola in particular (searched in August 2025)

No.	Search string	Number and percentage of compliant entries in various databases (n, %)			
		PubMed	Web of Science	Scopus	Europe PMC
1	Genetically modified plants AND hybrid AND (wild plants OR conventional varieties)	1128 (13 %)*	202 (33 %)	3883 (NA)	2464 (NA)
2	Genetically modified plants AND hybrid AND sexual crossing AND (wild plants OR conventional varieties)	5 (2, 40 %)	9 (0 %)	168 (NA)	276 (6 %)
3	(Genetically modified plant*) AND hybrid AND (sexual cross*) AND ((wild plant*) OR (conventional variet*))	5 (2, 40 %)	0	942 (NA)	1642 (NA)
4	((Genetically modified plant*) OR (transgenic plant*)) AND hybrid AND (sexual cross*) AND ((wild plant*) OR (conventional variet*))	6 (3, 30%)	0	2285 (NA)	2229 (NA)
5	Genetically modified potato AND hybrid AND (wild plant OR conventional varieties)	32 (1, 3.13 %)	13 (4, 30.77%)	1629 (NA)	739 (NA)
6	Genetically modified potato AND hybrid AND sexual crossing AND (wild plant OR conventional varieties)	0	5 (4, 80 %)	85 (7, 8.24 %)	115 (13 %)
7	(Genetically modified potato) AND hybrid AND (sexual cross*) AND ((wild plant*) OR (conventional variet*))	0	0	332 (NA)	345 (7 %)
8	((Genetically modified potato) OR (transgenic potato)) AND hybrid AND (sexual cross*) AND ((wild plant*) OR (conventional variet*))	0	0	344 (NA)	455 (5 %)
9	(Genetically modified plant OR GM plant) AND (oilseed rape OR *B. napus*) AND (herbicide resistance OR glyphosate resistance) AND (seed spillage OR transport OR monitoring)	21 (20, 95.24 %)	18 (100 %)	509 (NA)	140 (42 %)

NA – not assessed. *- First 100 entries grouped chronologically starting from the oldest were detailly assessed. The results are obtained by extrapolation from the first 100 entries.

Table S5. Search strings for NGTs elaborated from Eckerstorfer et al. (2019) [56] (searched in June - August 2025)

No.	Search string	Number and percentage of compliant entries in various databases (n)			
		PubMed	Web of Science	Scopus	Europe PMC
1.1	(crispr OR Cas9) AND (plant OR plants OR plant* OR "plant breeding" OR crop* OR tree*) AND hybrid AND (wild plants OR conventional varieties)	116 (11 %)	101	3028	1936 (9 %)
1.2.	(crispr OR Cas9) AND (plant OR plants OR plant* OR "plant breeding" OR crop* OR tree*) AND hybrid AND (wild plants OR conventional varieties) AND canola	3 (0 %)	0	285	159 (17 %)
1.3.	(crispr OR Cas9) AND (plant OR plants OR plant* OR "plant breeding" OR crop* OR tree*) AND hybrid AND sexual crossing AND (wild plants OR conventional varieties)	1 (0%)	100	90	22 (4, 18.18 %)
2.1.	(crispr OR Cas9) AND (plant OR plants OR plant* OR "plant breeding" OR crop* OR tree*) AND hybrid AND "sexual cross*" AND ("wild plant*" OR "conventional variet*")	0	0	0	0
2.2.	(crispr OR cpf1) AND (plant OR plants OR plant* OR "plant breeding" OR crop* OR tree*) AND hybrid AND (wild plants OR conventional varieties)	104 (8%)	98	2994	1909
2.3.	(crispr OR cpf1) AND (plant OR plants OR plant* OR "plant breeding" OR crop* OR tree*) AND hybrid AND sexual crossing AND (wild plants OR conventional varieties)	1 (0 %)	2	88	173
3.1.	(crispr OR cpf1) AND (plant OR plants OR plant* OR "plant breeding" OR crop* OR tree*) AND hybrid AND "sexual cross*" AND ("wild plant*" OR "conventional variet*")	0	0	0	0
3.2.	("transcription activated-like nuclease*" OR TALEN OR "transcription activator-like effector nuclease*") AND (plant* OR crop*) AND hybrid AND (wild plants OR conventional varieties)	3 (1, 33.33%)	1	509	222
3.3.	("transcription activated-like nuclease*" OR TALEN OR "transcription activator-like effector nuclease*") AND (plant* OR crop*) AND hybrid AND sexual crossing AND (wild plants OR conventional varieties)	0	0	17	34
4.1.	("transcription activated-like nuclease*" OR TALEN OR "transcription activator-like effector nuclease*") AND (plant* OR crop*) AND hybrid AND "sexual cross*" AND ("wild plant*" OR "conventional variet*")	0	0	0	0
4.2.	("zinc finger nuclease" OR ZFN) AND (plant* OR crop*) AND hybrid AND (wild plants OR conventional varieties)	0	0	483	155
4.3.	("zinc finger nuclease" OR ZFN) AND (plant* OR crop*) AND hybrid AND sexual crossing AND (wild plants OR conventional varieties)	0	0	28	20
5.1.	("zinc finger nuclease" OR ZFN) AND (plant* OR crop*) AND hybrid AND "sexual cross*" AND ("wild plant*" OR "conventional variet*")	0	0	0	0
5.2.	(oligonucleotid* OR "oligonucleotide directed mutagenesis" OR ODM OR "chimeric oligonucleotid*" OR "chimeric RNA/DNA oligonucleotid*" OR chimeraplasty OR "site-directed mutagenesis" OR "gene targeting") AND (plant* OR crop*) AND hybrid AND (wild plants OR conventional varieties)	687 (3 %)	27	1944	1112
5.3.	(oligonucleotid* OR "oligonucleotide directed mutagenesis" OR ODM OR "chimeric oligonucleotid*" OR "chimeric RNA/DNA oligonucleotid*" OR chimeraplasty OR "site-directed mutagenesis" OR	0	0	72	110

No.	Search string	Number and percentage of compliant entries in various databases (n)			
		PubMed	Web of Science	Scopus	Europe PMC
	"gene targeting") AND (plant* OR crop*) AND hybrid AND sexual crossing AND (wild plants OR conventional varieties)				
6.1.	(oligonucleotid* OR "oligonucleotide directed mutagenesis" OR ODM OR "chimeric oligonucleotid*" OR "chimeric RNA/DNA oligonucleotid*" OR chimeraplasty OR "site-directed mutagenesis" OR "gene targeting") AND (plant* OR crop*) AND hybrid AND "sexual cross*" AND ("wild plant*" OR "conventional variet*")	0	0	0	0
6.2.	"multiplex automated genomic engineering" AND hybrid AND (wild plants OR conventional varieties)	0	0	0	0
6.3.	"multiplex automated genomic engineering" AND hybrid AND sexual crossing AND (wild plants OR conventional varieties)	0	0	0	0
7.1.	"multiplex automated genomic engineering" AND hybrid AND "sexual cross*" AND ("wild plant*" OR "conventional variet*")	0	0	0	0
7.2.	(TGS) AND (plant* OR crop*); (RDDM OR RNA*directed DNA methylation) AND (plant* OR crop*) AND hybrid AND (wild plants OR conventional varieties)	0	0	0	71
7.3.	(TGS) AND (plant* OR crop*); (RDDM OR RNA*directed DNA methylation) AND (plant* OR crop*) AND hybrid AND sexual crossing AND (wild plants OR conventional varieties)	0	0	0	12
8.1.	(TGS) AND (plant* OR crop*); (RDDM OR RNA*directed DNA methylation) AND (plant* OR crop*) AND hybrid AND "sexual cross*" AND ("wild plant*" OR "conventional variet*")	0	0	0	0
8.2.	(cisgen* OR intragen* OR "all native DNA transformation" OR "all-native DNA transformation") AND (plant* OR crop*) AND hybrid AND (wild plants OR conventional varieties)	25 (7, 28%)	11	836	373
8.3.	(cisgen* OR intragen* OR "all native DNA transformation" OR "all-native DNA transformation") AND (plant* OR crop*) AND hybrid AND sexual crossing AND (wild plants OR conventional varieties)	0	0	68	82
9.1.	(cisgen* OR intragen* OR "all native DNA transformation" OR "all-native DNA transformation") AND (plant* OR crop*) AND hybrid AND "sexual cross*" AND ("wild plant*" OR "conventional variet*")	0	0	1	0
9.2.	graft* AND (transg* OR transform* OR GM graft OR GM scion) AND (plant* OR crop* OR tree*) AND hybrid AND (wild plants OR conventional varieties)	11 (0 %)	13	146	208
9.3.	graft* AND (transg* OR transform* OR GM graft OR GM scion) AND (plant* OR crop* OR tree*) AND hybrid AND sexual crossing AND (wild plants OR conventional varieties)	1 (0 %)	1	5	30
10.1.	graft* AND (transg* OR transform* OR GM graft OR GM scion) AND (plant* OR crop* OR tree*) AND hybrid AND "sexual cross*" AND ("wild plant*" OR "conventional variet*")	0	0	0	0
10.2.	(graft* OR transgraft* OR trans-graft*) AND ("GM rootstock*" OR "transgen* rootstock") AND hybrid AND (wild plants OR conventional varieties)	0	1	0	7
10.3.	(graft* OR transgraft* OR trans-graft*) AND ("GM rootstock*" OR "transgen* rootstock") AND hybrid AND sexual crossing AND (wild plants OR conventional varieties)	0	0	1	1

No.	Search string	Number and percentage of compliant entries in various databases (n)			
		PubMed	Web of Science	Scopus	Europe PMC
11.1.	(graft* OR transgraft* OR trans-graft*) AND ("GM rootstock*" OR "transgen* rootstock") AND hybrid AND "sexual cross*" AND ("wild plant*" OR "conventional variet*")	0	0	0	0
11.2.	(agroinfiltr* OR agroinocul* OR agroinfect*) AND (plant* OR crop*) AND hybrid AND (wild plants OR conventional varieties)	12 (0 %)	9	195	265
11.3.	(agroinfiltr* OR agroinocul* OR agroinfect*) AND (plant* OR crop*) AND hybrid AND sexual crossing AND (wild plants OR conventional varieties)	1 (0 %)	0	8	19
12.1.	(agroinfiltr* OR agroinocul* OR agroinfect*) AND (plant* OR crop*) AND hybrid AND "sexual cross*" AND ("wild plant*" OR "conventional variet*")	0	0	0	0
12.2.	(CENH3 OR "haploid induction" OR "genome elimination" OR haploids) AND (plant* OR crop*) AND hybrid AND (wild plants OR conventional varieties)	98 (1, 1.02)	122	3558	369
12.3.	(CENH3 OR "haploid induction" OR "genome elimination" OR haploids) AND (plant* OR crop*) AND hybrid AND sexual crossing AND (wild plants OR conventional varieties)	4 (0 %)	5	272	87
13.1.	(CENH3 OR "haploid induction" OR "genome elimination" OR haploids) AND (plant* OR crop*) AND hybrid AND "sexual cross*" AND ("wild plant*" OR "conventional variet*")	0	0	3	0
13.2.	("reverse breeding" OR "crossover control") AND (plant* OR crop*) AND (plant* OR crop*) AND hybrid AND (wild plants OR conventional varieties)	1 (0 %)	2	45	30
13.3.	("reverse breeding" OR "crossover control") AND (plant* OR crop*) AND (plant* OR crop*) AND hybrid AND sexual crossing AND (wild plants OR conventional varieties)	0	0	6	12
13.4.	("reverse breeding" OR "crossover control") AND (plant* OR crop*) AND (plant* OR crop*) AND hybrid AND "sexual cross*" AND ("wild plant*" OR "conventional variet*")	0	0	0	0
14.	experimental evidence about gene flow from NGT canola or potato field trials	442 (3, 3.00 %)	1877	2	236

Table S6. Search strings in PubMed and Europe PMC using controlled vocabulary thesaurus of Medical Subject Headings (MeSH):

1) "Plant Breeding"[MeSH Terms] AND "Plants"[MeSH Terms] AND "plants, genet-ically modified"[MeSH Terms] AND "hybridization, genetic"[MeSH Terms] AND "gene transfer, horizontal"[MeSH Terms] AND "Cotyledon"[MeSH Terms] – 0;

2) "Plants"[MeSH Terms] AND "plants, genetically modified"[MeSH Terms] AND "hybridization, genetic"[MeSH Terms] AND "gene transfer, horizontal"[MeSH Terms] AND "Cotyledon"[MeSH Terms] – 0;

3) "Plants"[MeSH Terms] AND "plants, genetically modified"[MeSH Terms] AND "hybridization, genetic"[MeSH Terms] AND "Cotyledon"[MeSH Terms] – 0;

4) "Plants"[MeSH Terms] AND "plants, genetically modified"[MeSH Terms] AND "hybridization, genetic"[MeSH Terms] – 307 (25 % of compliant entries) (Europe PubMed Central (Europe PMC) – 3);

5) "Plants"[MeSH Terms] AND "plants, genetically modified"[MeSH Terms] AND ("hybridization, genetic"[MeSH Terms] OR "Gene Flow"[MeSH Terms]) – 437 (Europe PMC – 6);

6) "Plants"[MeSH Terms] AND "plants, genetically modified"[MeSH Terms] AND "hybridization, genetic"[MeSH Terms] AND "Gene Flow"[MeSH Terms]) AND (re-view[Filter] – 56 (6 review) (Europe PMC – 1).

Figure S1. PRISMA flow diagram for the searches of databases

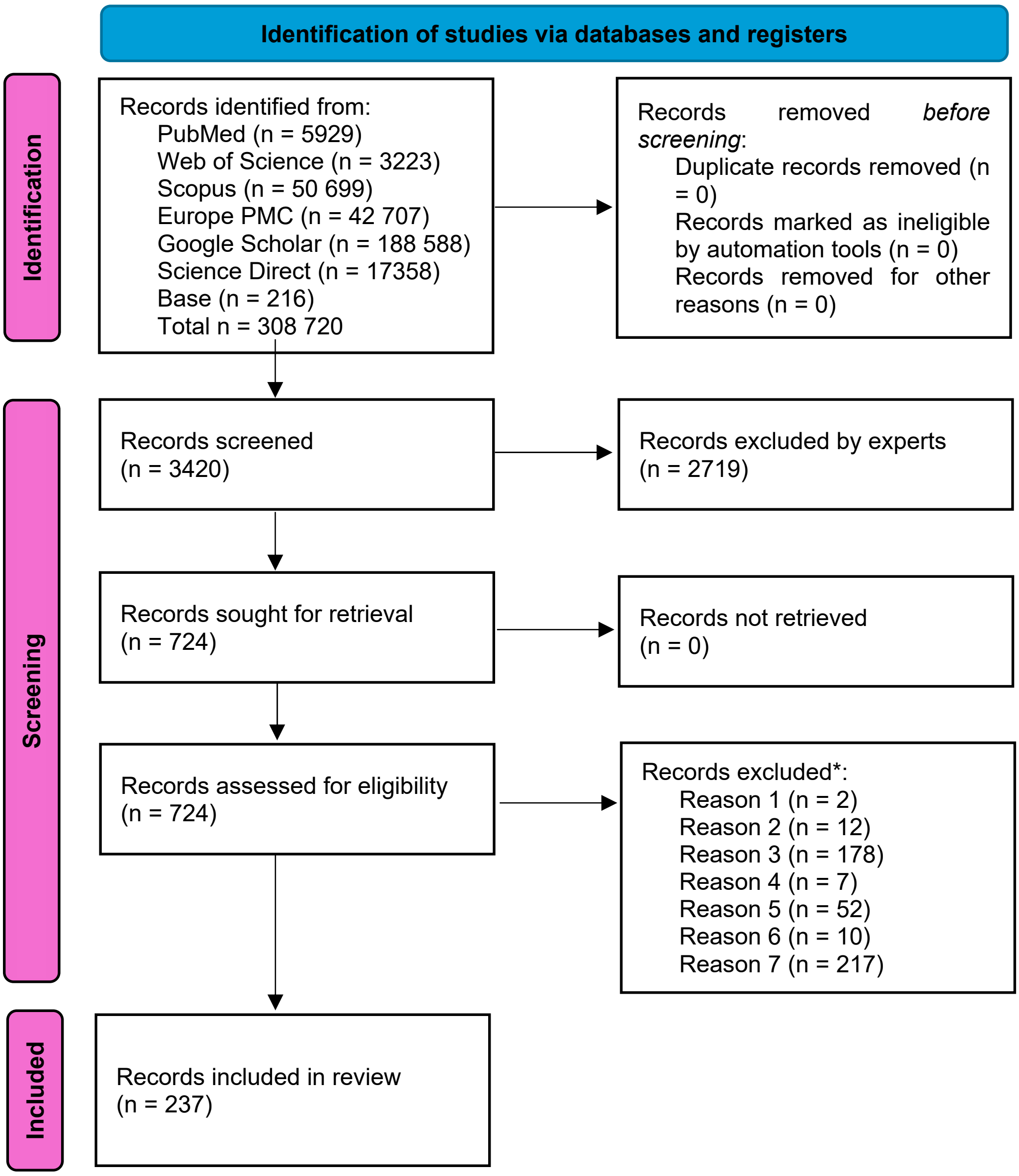

*Reason 1: Different topic (methodological / unrelated focus)

Reason 2: Not eligible by title, abstract, and keywords. Full text inaccessible

Reason 3: No hybridisation / gene flow content

Reason 4: Non-target species or non-GM material

Reason 5: Eligible by title, abstract, and keywords. As full text was not available, was not included in the literature review

Reason 6: Not eligible according to the opinion of the other expert

Reason 7: Review article, no new information etc.

Source: Page MJ, et al. BMJ 2021;372:n71. doi: 10.1136/bmj.n71.
This work is licensed under CC BY 4.0. To view a copy of this license, visit https://creativecommons.org/licenses/by/4.0/
